# Ectopic Expression of *Arabidopsis thaliana zDof1.3* in Tomato (*Solanum lycopersicum* L.) Is Associated with Improved Greenhouse Productivity and Enhanced Carbon and Nitrogen Use

**DOI:** 10.3390/ijms231911229

**Published:** 2022-09-23

**Authors:** Kietsuda Luengwilai, Jingwei Yu, Randi C. Jiménez, Maysaya Thitisaksakul, Andrea Vega, Shaoyun Dong, Diane M. Beckles

**Affiliations:** 1 Department of Plant Sciences, University of California, One Shields Avenue, Davis, CA 95616, USA; 2 Millennium Nucleus for the Development of Super Adaptable Plants (MN-SAP), Center of Applied Ecology and Sustainability (CAPES), Faculty of Engineering and Sciences, Universidad Adolfo Ibáñez, Santiago 7941169, Chile

**Keywords:** Dof, transcription factor, tomato, carbohydrates, nitrogen metabolism

## Abstract

A large collection of transgenic tomato lines, each ectopically expressing a different *Arabidopsis thaliana* transcription factor, was screened for variants with alterations in leaf starch. Such lines may be affected in carbon partitioning, and in allocation to the sinks. We focused on ‘L4080’, which harbored an *A. thaliana zDof* (*DNA-binding one zinc finger*) *isoform 1.3* (*AtzDof1.3*) gene, and which had a 2–4-fold higher starch-to-sucrose ratio in source leaves over the diel (*p* < 0.05). Our aim was to determine whether there were associated effects on productivity. L4080 plants were altered in nitrogen (N) and carbon (C) metabolism. The N-to-C ratio was higher in six-week-old L4080, and when treated with 1/10 N, L4080 growth was less inhibited compared to the wild-type and this was accompanied by faster root elongation (*p* < 0.05). The six-week-old L4080 acquired 42% more dry matter at 720 ppm CO_2_, compared to ambient CO_2_ (*p* < 0.05), while the wild-type (WT) remained unchanged. GC-MS-TOF data showed that L4080 source leaves were enriched in amino acids compared to the WT, and at 49 DPA, fruit had 25% greater mass, higher sucrose, and increased yield (25%; *p* < 0.05) compared to the WT. An Affymetrix cDNA array analysis suggested that only 0.39% of the 9000 cDNAs were altered by 1.5-fold (*p* < 0.01) in L4080 source leaves. ^14^C-labeling of fruit disks identified potential differences in 14-DPA fruit metabolism suggesting that post-transcriptional regulation was important. We conclude that *AtzDof1.3* and the germplasm derived therefrom, should be investigated for their ‘climate-change adaptive’ potential.

## 1. Introduction

The tomato (*Solanum lycopersicum* L.) is one of the most widely consumed and cultivated crops, forming an important market on nearly every continent [1]. Optimizing tomato yield by producing plants that require lower chemical inputs, without compromising productivity would improve the sustainable cultivation of this crop, especially in the face of global climate change [2]. Yield, growth, and productivity are complex traits determined by the interaction of a multitude of spatially and temporally separated events [3,4], which are encoded by multiple genes, the actions of which are strongly influenced by environmental conditions [5]. At the physiological level, they regulate the uptake of assimilates in leaves (carbon) and roots (nitrogen) [6,7], thus supporting the number of inflorescences as well as later events during fruit establishment such as fruit cell division, expansion, import, and subsequent metabolic processes [8,9]. Still, quantitative traits and single loci that promote higher yield in tomato have been identified [4,7,10,11,12,13,14], suggesting that there may be other key genes that can exert measurable effects on important agronomic traits.

Transcription factors (TFs) act as master regulators of gene expression by activating or repressing transcription or by regulating miRNAs [15,16]. The cohort of genes they may regulate in tandem usually comprise a common signaling, regulatory, or metabolic pathway [15,17,18,19]. Many aspects of tomato growth, development and yield are controlled by TFs [18]. These processes include the regulation and integration of general metabolism, plant architecture, and the mobilization of protective mechanisms to mitigate stress [18,20,21,22].

As part of an attempt to modify economically important traits in tomato, a large-scale functional genomics platform was implemented to clone individual transcription factors from *Arabidopsis thaliana* (L.) Heynh, and to transform and ectopically express them in individual tomato lines [18]. This was a forward genetic screen that emphasized genotypes with interesting agronomic traits [18,23]. We screened this population for genotypes mis-regulated in carbon allocation and partitioning, which may influence yield and productivity [23,24]. This prescreen was accomplished by assessing source leaves for differences in leaf starch. Our hypothesis was that if starch is a key ‘integrator’ of growth and development [23], then lines varying in leaf starch could also be altered in yield and productivity.

Here, we report a limited analysis of a transgenic tomato line identified from this screen. The line, so-named L4080, ectopically expressed *AtzDof1.3* (*A. thaliana DNA-binding one zinc finger 1.3*; locus AT1G26790) [25]. Some *zDof* genes have been implicated in the regulation of primary metabolism by co-expression with genes involved in carbon and nitrogen metabolism in maize [26], wheat [27], and millet [28,29]. Furthermore, overexpression or suppression of native genes, or the ectopic expression of some *zDof* orthologues in *A. thaliana* [30,31,32], tomato [33,34], soybean [35], rice [36], tobacco [37], canola [38] and sweet potato [39] led to changes in carbon metabolism, nitrogen metabolism or both, suggesting direct involvement of some *zDofs* in coordinating the regulation of these pathways.

The primary aim of this work was to determine how line L4080 compensated for the shift in source leaf carbohydrates, and to determine whether there were consequences in its growth and agronomic performance under greenhouse conditions. We wish to understand how the plant responds at all levels by using combined eco-physiology, metabolomics, and transcriptomic analyses. Attention was paid to nitrogen and carbon allocation because of their importance in determining crop yield and resilience, and evidence that some *zDofs* may regulate some aspects of these metabolic processes [33,34,39,40,41,42]. The second aim was to take advantage of growing genomic sequence resources to learn more about the evolutionary relatedness of *AtzDof1.3* to other *A. thaliana zDofs*, and orthologues of tomato and other dicots. Further, determining the relationships of *AtzDof1.3* homologues in cultivated and wild tomatoes could help determine whether the diversification of this gene was a target during crop selection and the potential for improvement in tomato.

## 2. Results

### 2.1. Selection and Initial Characterization of the Transgenic Line

The tomato line investigated was selected by screening a large collection of diverse transgenic tomato genotypes. Each line was transformed with two constructs, one bearing one of the 1733 Arabidopsis transcription factors, and the other, one of five different promoters, which directed either constitutive or tissue-specific gene expression [43]. The hypothesis was that TFs integrating whole plant carbon allocation with growth could be selected by screening the mutants for alterations in leaf starch [24,44]. A pre-screen of leaf starch content was conducted on ~7000 field-grown plants. From this, ~30 lines which deviated significantly in leaf carbohydrate content from the transgenic (transformed with an empty vector) and untransformed controls (*p* < 0.05), were grown in the greenhouse. Leaf and fruit were characterized for alterations in sugars and starch at different developmental stages. The line tagged as L4080 was selected since this secondary screen suggested that it accumulated 2-fold higher sucrose in leaves than the control. In addition, published data pointed to an important role for *A. thaliana zDof* homologues in C-N regulation and it was of interest to determine whether the results would be recapitulated in a crop plant such as tomato [30].

To verify that L4080 harbored the designated constructs in its genome, primers were designed to the regions flanking the engineered promoter and TF, and were used to amplify these segments by PCR. BLAST analysis of the sequences identified the transgenes as the tomato phytoene desaturase promoter and the *DNA-binding one zinc finger protein isoform 1.3* from *A. thaliana* (*AtzDof 1.3*; gene locus AT1G26790). This *A. thaliana* gene also described as CYCLING DOF FACTOR 6, is controlled by a circadian clock, and has known roles in *A. thaliana* flowering, and development under stress [45,46]. Transcripts of the heterologous *AtDof1.3* and the actin control were co-amplified in sink and source leaves and flowers, but surprisingly, given that the transgene was driven by phytoene desaturase, only actin, not *AtzDof1.3*, was easily detected in fruit at three developmental stages under the conditions employed (Supplementary Figure S1). When the untransformed control was examined, only actin, and not *AtzDof 1.3* was detected.

### 2.2. Sugar and Starch Accumulation and Partitioning in Leaf and Fruit

Leaf carbohydrates were assayed with the primary focus on starch and sucrose as their relative abundance are indicative of plant carbon and energy status [47,48]. Alterations in their levels in L4080 compared to the wildtype may indicate diversions in the strategy for resource allocation, with consequences for plant development [24,44].

**Figure 1:**
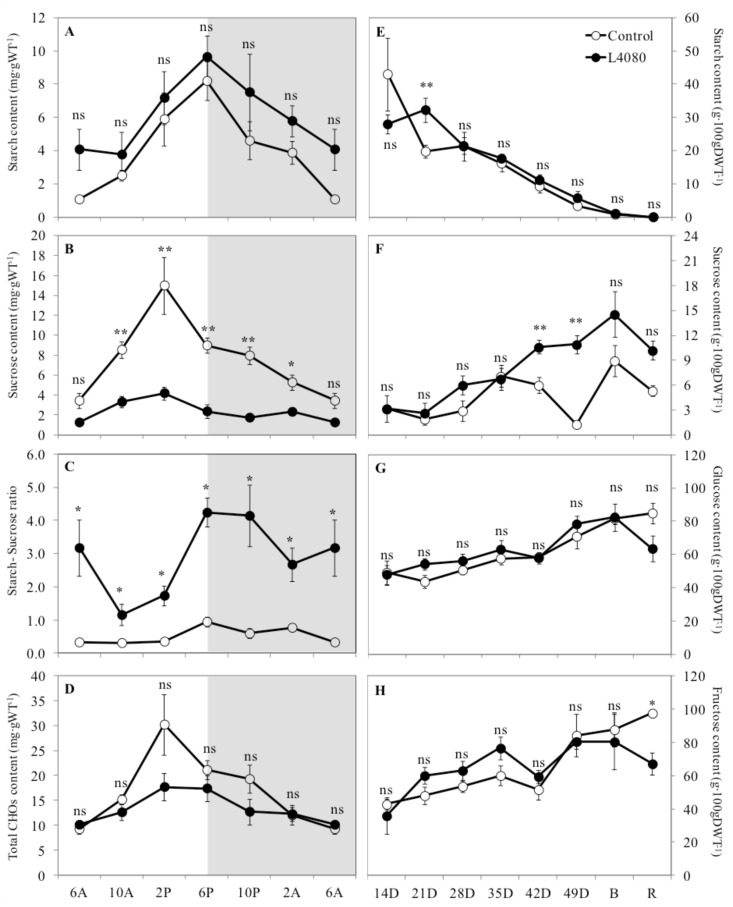
Carbohydrate content in control and transgenic tomato line L4080. (1) Changes in leaf carbohydrate content (g.DWT^−1^) during the diel. (**A**) Starch, (**B**) Sucrose, (**C**) Starch-to-Sucrose ratio and (**D**) Total carbohydrates. The latter is the sum of starch, sucrose, glucose, and fructose contents. The data shown for 6 AM were presented twice to better illustrate the trend over 24 h. The shaded area represents the night period. (2) Fruit carbohydrates assayed in the pericarp taken at 7-day intervals during fruit development. The graphs indicate the following compounds: (**E**) Starch, (**F**) Sucrose, (**G**) Glucose and (**H**) Fructose. DPA—days post anthesis, B—fruit at Breaker stage ~63 DPA, and R—fruit at red ripe ~70 DPA. Data are the mean ± SEM of 6 biological replicates. * and ** indicates that the data points differ between genotypes at *p* < 0.05 and *p* < 0.01, respectively. ‘ns’ indicates no significance difference, i.e., *p* > 0.05.

*Source leaves.* L4080 accumulated 2–4-fold less sucrose throughout the day except at the end of the night/beginning of the day, leading to a higher starch-to-sucrose ratio (2–3-fold) at every time point assayed (Figure 1). The biggest disparity between the genotypes was in the middle of the light period (Figure 1A). Total carbohydrates, i.e., the additive amount of starch, sucrose, glucose, and fructose, were the same between the control line and L4080 (Figure 1D).

Differences in the leaf starch-to-sucrose ratio may be due to variation in CO_2_ uptake and subsequent metabolism in L4080. Therefore, disks isolated from source leaves were fed with ^14^CO_2_ and total ^14^C-uptake and allocation to different bulk fractions of major biomolecules were monitored [49]. However, there were no detectable differences in either the amount, the rate of uptake or allocation of ^14^C into leaf sugars or starch. Only rapid metabolic changes would be detected using this method, and such modifications may not underscore the divergent leaf starch and sucrose in the two genotypes. It is also possible that the ^14^CO_2_ concentration was high and saturated the tissues, so that no differences could be detected.

*Developing fruit.* When fruit carbohydrate content (g.dwt^−1^) was expressed on a developmental index to account for differences in growth due to the genetic perturbation [50], there was no significant difference in starch, sucrose, glucose, or fructose between genotypes. However, when the data were plotted as days post anthesis (DPA), L4080 accumulated more starch at 21 DPA (Figure 1E) and more sucrose at 42 and 49 DPA compared to the control (Figure 1F).

To obtain a more dynamic picture of fruit metabolism, pericarp disks were fed ^14^C-glucose in an in vitro system [50,51,52] using fruit of the same age as those used to assay carbohydrates in Figure 1. ^14^C-partitioning data [53] are a better measure of the rate of synthesis of a compound, compared to steady-state measurements, which reflect the net balance of synthesis, minus degradation [51]. Across development, the sugar pool was the most dynamic, with higher proportions of ^14^C-in L4080 at 28 DPA and in red fruit (Figure 1). There was also an inverse correlation between the starch–sugar flux in different stages, i.e., when ^14^C in sugars was high in either genotype, ^14^C in starch would be low and vice versa.

**Figure 2:**
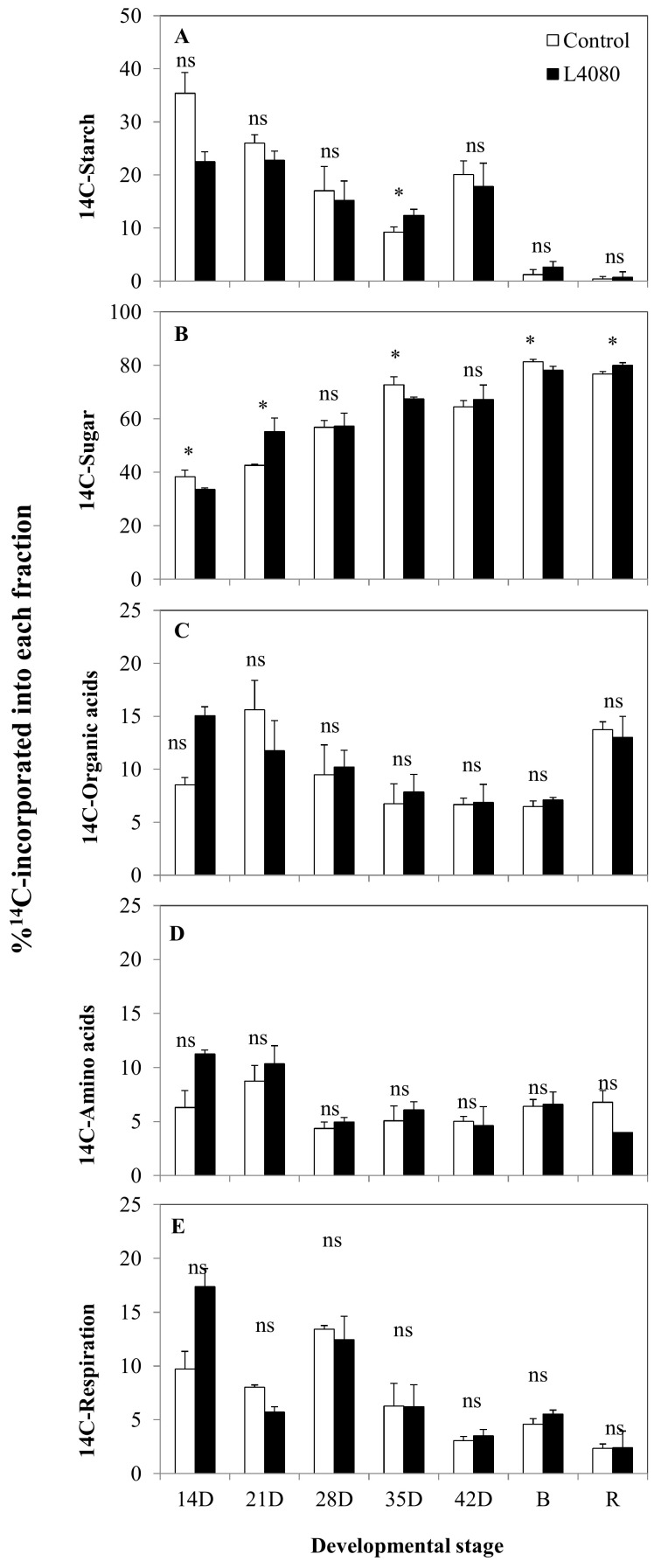
An in vitro study of carbon partitioning in developing tomato fruit using ^14^C-glucose labeling. Disks sampled from the pericarp of tomato fruit at varying developmental stages were fed with ^14^C-glucose and the percentage of ^14^C that partitioned into various fractions was compared between genotypes. Fractions examined were (**A**) Starch, (**B**) Sugars, (**C**) Organic acids, (**D**) Amino acids and (**E**) Respiration. Data are the mean ± SD of 3 biological replicates. An asterisk indicates data points differing between genotypes (*p* < 0.05), while ‘ns’ indicates values that did not reach this criterion.

The partitioning of ^14^C-glucose into starch and sugar did not always correlate with net sugar and starch accumulation, shown in Figure 1, Figure 2 and Supplementary Figure S1. These asynchronous metabolic events may be due to differences in the cycles of synthesis and degradation of the respective metabolic pools in tomato fruit [50,51,54]. For example, more ^14^C was partitioned into starch in L4080 at 35 DPA compared to the control (Figure 2A), while net starch accumulation was identical between genotypes at this stage (Figure 1E) [51,52]. This could be explained if the rate of starch degradation in L4080 was higher compared to the wild-type [51,52]. Likewise, the ^14^C-label that partitioned into sugars was lower in L4080 at 14, 35 DPA and at breaker (Figure 2), but net sugar accumulation was the same in the genotypes (Supplementary Figure S2), indicating higher sugar degradation rates or conversion to other compounds in the control fruit.

It was also interesting to note that ^14^C-allocation into amino acids, organic acids, and respiratory activity (Figure 2C–E) were higher in L4080 in very young fruit (14 DPA), although the differences were not significant (*p* > 0.05).

### 2.3. Plant Productivity under Limited Nitrogen and Higher Carbon Dioxide (CO_2_)

**Figure 3:**
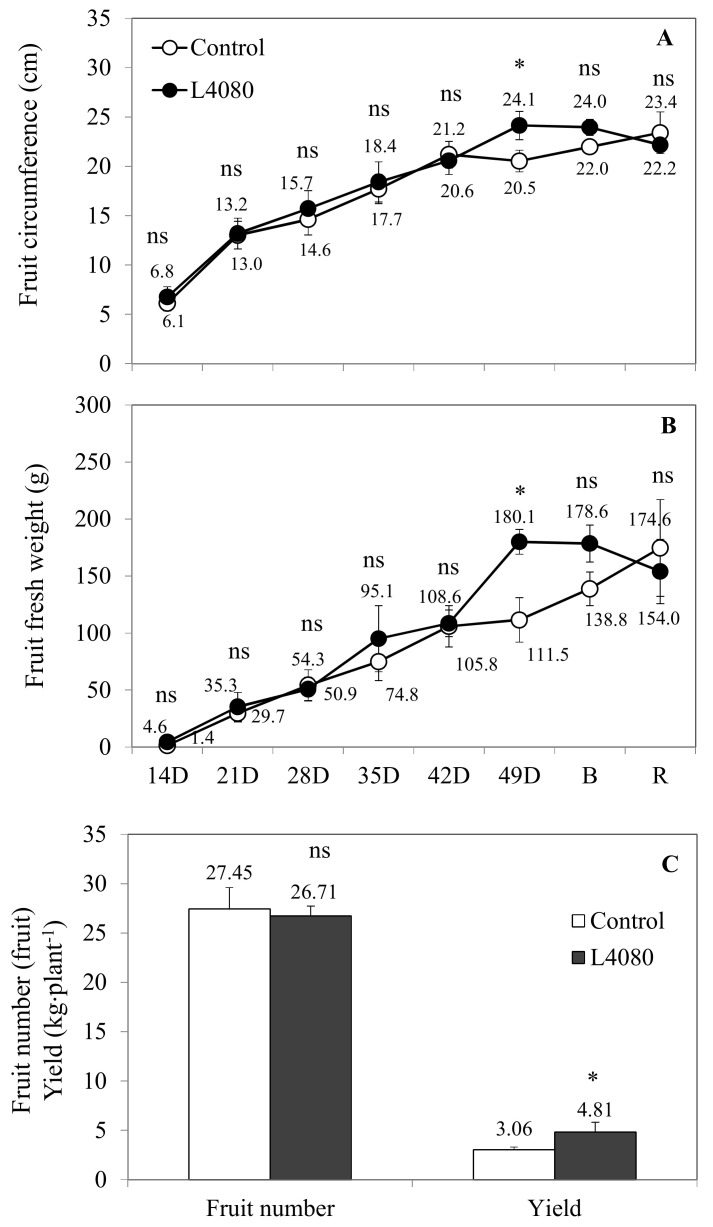
Measures of plant productivity assessed by fruit characteristics during development. Fruit (**A**) circumference, (**B**) fresh weight and number, were determined from fruit on trusses 1–9, using additional plants. (**C**) Yield data (fruit number × fruit fresh weight) were collected at 49 DPA and are the Mean ± SD of six different plants. The asterisk indicates data points differing between genotypes at *p* < 0.05, while ‘ns’ indicates values that did not reach this criterion.

Yield is the ultimate indicator of agronomic productivity. Fruit mass, circumference, and fruit numbers are critical parameters of yield, and these were assayed throughout fruit development. At 49 DPA, which represents physiological maturation, yield was 25% higher (*p* < 0.05) in L4080, attributable to 25% higher fruit fresh mass, since the fruit number was similar (Figure 3).

Other members of the *zDof* transcription factor family, when overexpressed, showed enhanced storage product accumulation [33,39,55,56]. The additional mass observed in L4080 was due to higher water content as the genotypes contained the same fruit dry matter content. Total soluble solids (TSS) of the red ripe fruit did not vary between cultivars and, thus, neither did horticultural yield (TSS × yield).

**Figure 4:**
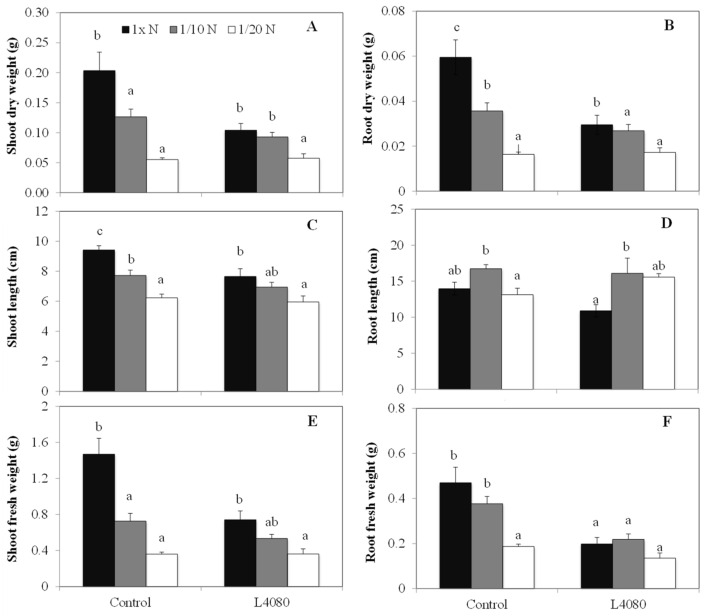
Growth characteristics under limited Nitrogen. Plants were grown in a controlled environment in vermiculite and supplemented with ½ strength Hoagland solution modified to vary in N-content. After 6-weeks, the roots and shoots were harvested, and their dry weights (**A**,**B**) lengths, (**C**,**D**), and fresh weights (**E**,**F**) assessed, respectively. Different letters indicate means that differ (*p* < 0.05) depending on N-concentration within a given genotype.

*Growth under limited N in a controlled environment chamber.* Members of the *zDof* gene family have been implicated in controlling some aspects of N-use in plants [27,30,57,58]. If *AtzDof1.3* is one such *zDof* and the proteins and its regulon are conserved between *Arabidopsis* and tomato, then L4080 would be expected to tolerate growth under lower N better than the control. Plants were grown in nutrient solutions with normal, 1/10 and 1/20 N. L4080 was lower in mass under normal N, but growth parameters in L4080 were not as perturbed by low N as the control line (Figure 4).

Shoot growth (gleaned from shoot length) in the control was reduced at 1/10 vs. normal N, (Figure 4A,C,E), while it was unchanged in L4080 (*p* < 0.05). Further, root biomass was better maintained, and root length increased at a faster rate under low N conditions in L4080 (*p* < 0.05) (Figure 4D,F) compared to the wild-type [59].

Equal amounts of dried tissue (shoot and root) from the two genotypes used in this experiment were analyzed for total N- and C-content. In L4080, N-content was higher than the wild-type under normal conditions, and the difference increased under 1/10 N (Supplementary Figure S4A). However, at 1/20 N, it was lower than wild-type. In contrast, as external N decreased, cellular N slowly increased in the wild-type (Supplementary Figure S4A). The N-to-C ratio was higher in L4080 (Supplementary Figure S4B), consistent with a higher N-use efficiency, which, in the simplest sense, describes the amount of N per unit mass of tissue [60].

We next determined whether there were differences in root characteristics in the transgenic line that may potentially facilitate more efficient root–soil interaction and uptake of N. Seedlings were grown on vermiculite in the experiment depicted in Figure 4 to enable easy root measurements. However, cultivation in soil may influence root growth and morphological dimensions [61], to the extent that there may be no differences observed between genotypes. To test this, plants were grown in 10-gallon pots to prevent them from becoming root-bound. After three-weeks of cultivation, taproots were longer (*p* < 0.05) in the transgenic line under normal N (Supplementary Figure S4). However, the data were the same for the genotypes as they advanced in development. The data suggest that the genetically altered line may not only metabolize N differently, but that this may be coupled with a more responsive, early root growth.

**Figure 5:**
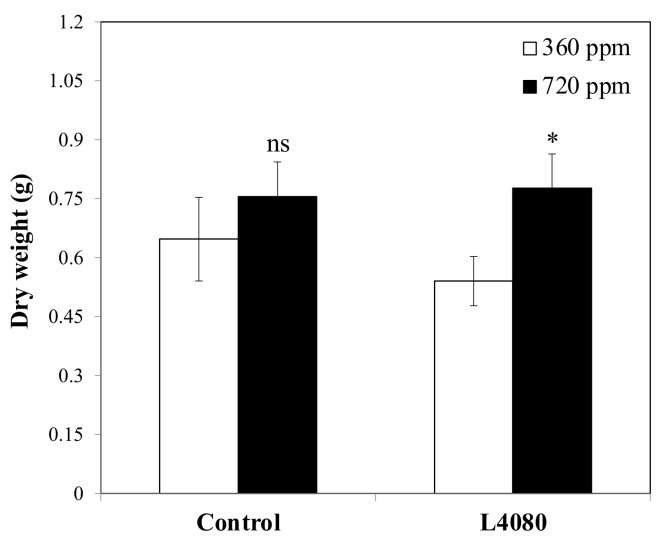
Response of seedlings to altered CO_2_. There were no significant differences in dry matter between the two genotypes under either condition. However, while the wild-type control did not change in biomass when exposed to elevated CO_2_, L4080 was able to accumulate 44% more dry matter. An asterisk indicates data points differing between genotypes (*p* < 0.05), while ‘ns’ indicates values that did not reach this criterion.

*Growth under high Carbon Dioxide (CO_2_).* C_3_ plants such as tomato may not be able to use higher CO_2_ for biomass increases without N-supplementation [62]. We hypothesized that L4080 may be able to overcome this limitation if it has higher cellular N or better N-uptake mechanisms. Similar to the greenhouse experiment, dry weight was not statistically different between genotypes under normal CO_2_ (*p* > 0.05; Figure 5). When grown under 720 ppm CO_2_, dry matter in the wild-type plants was unchanged (*p* > 0.05). In contrast, L4080 dry weight was 43% higher under elevated CO_2_ (*p* = 0.03). The basis for this increase is not known, but it is possible that the higher endogenous N in L4080 led to more efficient metabolism and conversion of the additional CO_2_.

### 2.4. Eco-Physiological Parameters

The growth and development of the tomato lines were monitored over their lifecycle and multiple data were collected. There were no differences in the rate of leaf growth, plant growth (change in plant height assayed from transplant until constant) and internode growth. The number of flowers and fruit were similar as was shoot and root dry weight in L4080 compared to the control, and phenotypically the plants were very similar (Supplementary Figure S6).

### 2.5. Metabolite Profiling of Leaf and Fruit

**Figure 6:**
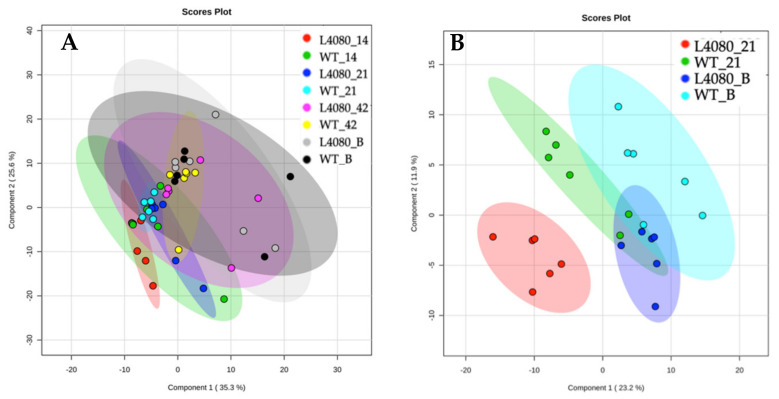
Relatedness of the GC-MS-TOF metabolite profiles of control and L4080 tissues fruit and leaf tissues. (**A**) A small disk of pericarp was sampled from the equator of fruit harvested at different developmental stages. The labels indicate: genotype, i.e., WT or L4080, followed by developmental stage i.e., 14, 21, 42 and B (Breaker ~49 DPA). (**B**) Leaves adjacent to fruit at 21 DPA and at Breaker.

GC-MS-TOF profiles of leaf and fruit extracts from the two lines were compared to provide a comprehensive overview of the various metabolic steps in each organ. Specifically, we wished to (a) gauge the extent to which there were perturbations in metabolism and (b) to pinpoint key steps that may explain differences in physiological characteristics between the genotypes. To gain an overview of the compositional differences in the genotypes, Partial Least Squares Discriminant Analysis (PLS-DA) of fruit and leaf metabolites was performed [63]. There was little or no distinction in the fruit metabolome between the two genotypes (Figure 6A).

This highlights the compositional similarity of the tissues despite any physiological divergence due to their differing age or genotype. Leaf tissue, however, showed some differences: 21-DPA L4080 leaf was an outlier, while the others all clustered together (Figure 6B).

The 112 individual metabolites leaves harvested adjacent to Breaker and 21-DPA fruit were inspected to identify genotype-specific differences (Table 1). In the L4080 younger leaves, 28% of the metabolites were altered, but few (6%) varied more than two-fold from the wild-type (*p* < 0.05). Breaker leaves had fewer changes (22% of total), but roughly 44% of this subset varied by two-fold or greater, and all but two were higher in L4080 (Table 1). Because of the evidence for differences in N-metabolism and uptake in L4080 and in published data [30,31,33,36,37], changes in the amino acid pool were noted. Of the 112 metabolites detected, 11 metabolites in leaf could be classified as amino acids or N-related compounds. At 21 DPA, five of the 11 amino acids (50%) were higher in L4080 compared to the WT, while in the 42 DPA leaves, the percentage was 72%, (8 of 11 amino acids) and were higher in L4080, consistent with changes in N-use in the transgenic line. Only three metabolites, all amino acids, were altered in leaves at both developmental stages, and each occupied important junctures in N-metabolism: aspartate, shikimate, and phenylalanine (Table 1) [64]. Aspartate is a hub for C- and N-metabolism, shikimate is the key branchpoint for the synthesis of aromatic amino acids and secondary metabolites, and phenylalanine is the substrate for diverse protective compounds [64].

Compared to leaf tissue, fewer metabolites (53) were detected in fruit, and there was minimal divergence in L4080 (Supplementary Table S1). L4080 21-DPA fruit showed the most dynamism, differing from the wild-type in 11 metabolites (21%), and interestingly, they were all suppressed (Supplementary Table S1). Three metabolites differed from the control in 42 DPA fruit, while only one metabolite differed in 14 DPA fruit and those of the breaker fruit were identical to wild-type. There was no clear pattern in the types of metabolites that were altered.

**Figure 7:**
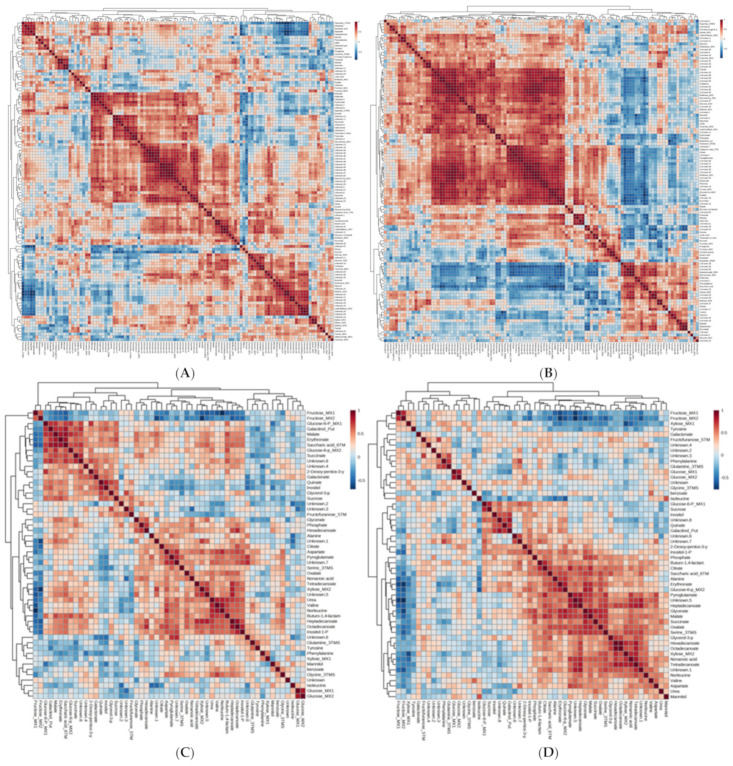
Heat maps showing correlative analysis of metabolites assayed by GC-MS-TOF in control and L4080 tomato fruit and leaves. Metabolite–metabolite correlations were calculated based on the combined GC-MS-TOF profiles of all tissues analyzed, 112 in leaf, and 52 in fruit and used to plot the above heat maps. (**A**) Wild-type and (**B**) L4080. Leaves harvested were either next to 21 DPA fruit or, breaker fruit for the two genotypes and these two datasets were combined for each genotype. The same was performed for (**C**) Wild-type and (**D**) L4080 developing fruit at 14, 21, 42 DPA and at breaker. The statistical significance of the correlations (Spearman’s Rank Coefficients) was not tested.

Although there were no major changes in the relative levels of metabolites in L4080, variations in how each metabolite correlated with all others across tissues could reveal broad and important underlying perturbations in L4080 metabolism compared to the control [63,65]. Without genetic or environmental perturbations, the relative levels of metabolites in a tissue should be reflected in near identical heatmaps. Metabolite-to-metabolite correlations for leaf and fruit tissues of the wild-type were depicted in heat maps and compared with those for L4080 (Figure 7). Fundamental alterations of the metabolic networks in L4080 are immediately evident, even in fruit, where the levels of individual metabolites did not vary (*p* < 0.05) from the control. Further, in leaf tissue, many of these changes in metabolite–metabolite associations were among amino acids, which formed novel and strong links with other metabolites in L4080 but not in the wild-type (Figure 7).

### 2.6. Transcriptomic Analysis of Leaf Tissue

The Affymetrix GeneChip v2.4 was used to probe differences in transcript abundance in the leaf tissue due to the genetic changes in L4080. Tissue was harvested 6 h after the start of the day consistent with the harvest time of other analyses. Of the 9245 transcripts on the array, only 39 or 0.39% differed between the control and L4080 by 1.5-fold or greater, when *p* ≤ 0.01 was used as the benchmark to assess statistically significant differences (Supplementary Table S2). Based on gene ontology annotations, eight cDNA sequences were of “unknown function”, 17 were involved in metabolism/catalytic activity, nine could be classified as transcription factors, while the remainder were receptors or genes of other functions (Supplementary Table S2).

A total of four cDNAs showed more than three-fold expression level changes in L4080. A ferric chelate reductase gene (*FRO1*) showed the greatest difference in transcript abundance (12-fold increase). *FRO1* is positively regulated in leaves of plants experiencing Fe-deficiency [66]. There is evidence for cross talk between the N- and Fe-signaling and metabolic pathways [67,68], evoking the possibility that the altered *Fro1* expression is connected to the higher N in L4080. The higher expression of the ATP/ADP transporter (5-fold) in L4080, could point to an altered energy status, as this transporter is essential for plastid acquisition of ATP at night [69]. *GA-2 oxidase isoform 7* was expressed 3.9-fold higher in L4080. This is a ‘gibberellin (GA) deactivating’ gene, which regulates meristematic functions [70]. Its expression in L4080 may reduce GA and alter growth. The threonine deaminase cDNA on the array was likely *TD1*, which bears a housekeeping function in producing the amino acid isoleucine [71]; its higher expression (3.4-fold) may modify the L4080 amino acid pool.

Of the known direct gene targets identified by various paralogues of the zDof transcription factors family [30,72] that were arrayed on this chip, most did not meet the criterion to be classified as being differentially expressed in L4080. Two phosphoenolpyruvate carboxylases, and two cDNAs involved in nitrite and nitrate regulation were only weakly differentially expressed (less than 2-fold) with *p*-values <0.05 (Supplementary Table S2). Further, the metabolomics and transcriptomics were performed on identically sampled 21 DPA leaves (Table 1 and Table S2). When the data were compared, there was no metabolite-to-gene connection, i.e., changes in gene expression that correlated with differences in cognate metabolites.

**Figure 8:**
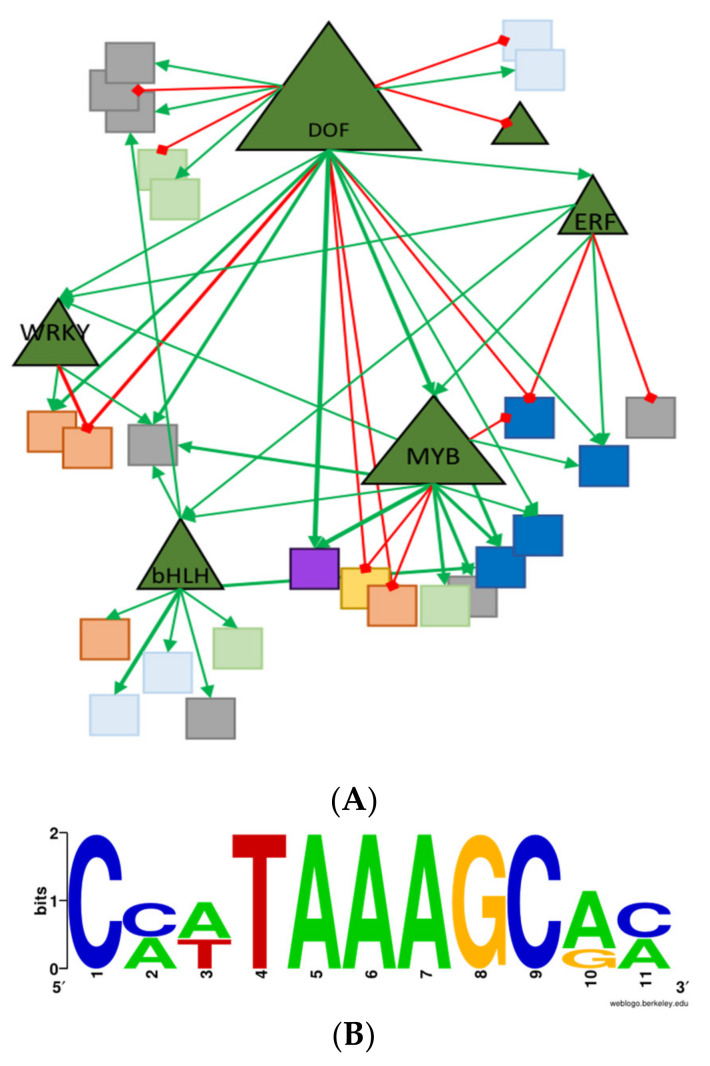
Network of promoter analysis and transcriptomics data of zDof-regulated genes. (**A**) Sequence of the *zDof*-motif located in the promoter from zDof-regulated genes. (**B**) Network of differentially expressed transcripts. The key is as follows: Triangles indicate transcription factors and squares denote the target genes. Green lines indicate predicted transcriptional activation (promoter analysis), whereas red lines indicate predicted transcriptional repression (promoter analysis). Thin lines indicate the level of gene expression. Arrows indicate positive regulation. Lines with a square at the end indicate negative regulation. Nodes are color-coded based on function: hormone (light blue), cell wall (orange), signaling (yellow), transport (purple), unknown genes (gray), protein and amino acid metabolism (blue).

A deeper analysis of the transcriptomic data was undertaken to potentially unravel layers of transcriptional regulation. The presence of known transcription factor binding sites in the 2000 bp genomic region upstream of the translational start codon for differentially expressed genes were searched (Supplementary Table S3). As expected, our analysis produced a significant enrichment in the [A/T]AAAG motifs in the promoter region of 20 *Dof*-regulated genes, compared to the average distribution in intergenic regions throughout the tomato genome (*p* < 0.05; Figure 8A,B). Among the remaining regulated genes, they contain mostly bHLH (basic Helix–Loop–Helix) and MADS motifs. Interestingly, a bHLH transcription factor was identified in the group of zDof-regulated genes (Figure 8).

MYB, NAC and C2H2 motifs were also identified in the promoter of 12 genes, suggesting that these genes could be regulated by other stimuli. An ERF and a WRKY transcription factor were also potentially induced by AtzDof.1.3, since four and five genes, respectively, contained DNA sequences in their promoter that could act as specific binding sites for these TFs. These observations suggest the possibility of a complex transcriptional regulatory network involved in C- and N-metabolism, in which several transcription factors act together.

### 2.7. Connecting The Novel Traits to AtzDof1.3 Expression

**Figure 9:**
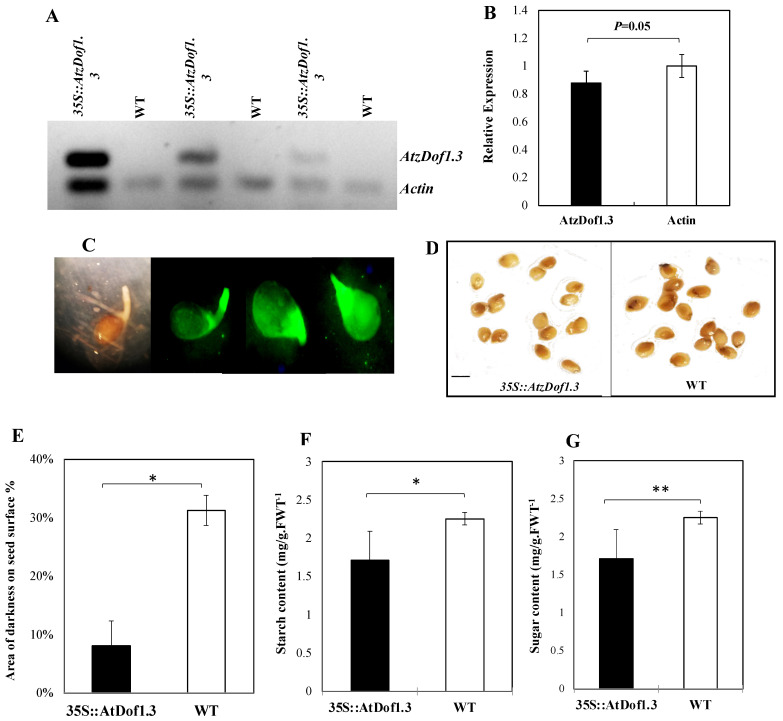
Transient expression of *AtzDof1.3* in tomato tissue. (**A**) RT-PCR products of *AtzDof1.3* and the actin control in transformed and WT seedling after gel electrophoresis. RNA of varying concentrations was loaded into each reaction (**B**) Equal expression of *AtzDof1.3* and actin the transformed line. (**C**) GFP fluorescence of transgenic tomato seeds transformed with the *AtDof1.3* construct (**D**) *AtDof1.3*–transformed and WT seeds stained with iodide and (**E**) their relative staining intensity. (**F**) Starch and (**G**) Sugar content of transformed lines compared to WT. * and ** indicate that the differences between the means are significant at *p* < 0.05 and *p* < 0.01, respectively. The scale bar in (**D**) is 0.5 mm.

All of the analyses described thus far were performed on only one variant identified through the screen. It is possible that the phenotype arose due to epigenetic modifications through somaclonal variation arising in tissue culture. Therefore, we cloned and sequenced the AtzDof1.3 cDNA from *A. thaliana*, and spliced it adjacent to a constitutive promoter and transiently expressed it in imbibed tomato seeds. The presence of GFP was confirmed indicating successful transformation of the construct (Figure 9A), and the *AtzDof1.3* transcript was detected in the GFP-positive line and not in the WT control (Figure 9B).

Further, the presence of the *AtzDof1.3* was associated with lighter iodide-staining in the seeds indicating altered starch metabolism (Figure 9C). This was confirmed when starch was assayed and found to be 24% lower in the transgenic tissue (Figure 9D). Moreover, the sugar content was lower (12%) consistent with a broader change in carbohydrate metabolism (Figure 9E). These data support several of the observations detected in L4080 identified through the screen.

### 2.8. Sequence, Evolutionary, and Comparative Analysis of AtzDof1.3

The *AtzDof1.3* gene ectopically expressed in tomato (AT1G26790) is a member of the Group D1 *zDof* TFs or “CYCLING DOF TFs”, so named because their transcripts oscillate in leaves even under constant light [73]. These TFs regulate flowering time and plant response to abiotic stress [73]. First, we asked how many of the known *zDofs* that led to changes in N, C, or biomass belonged to this group. Our review shows that of the ten such *zDofs* identified, only one, *SRF1*, was a D1-type *zDof* (Supplementary Table S4), while the other genes belonged to the A-, B-, and C-group.

**Figure 10:**
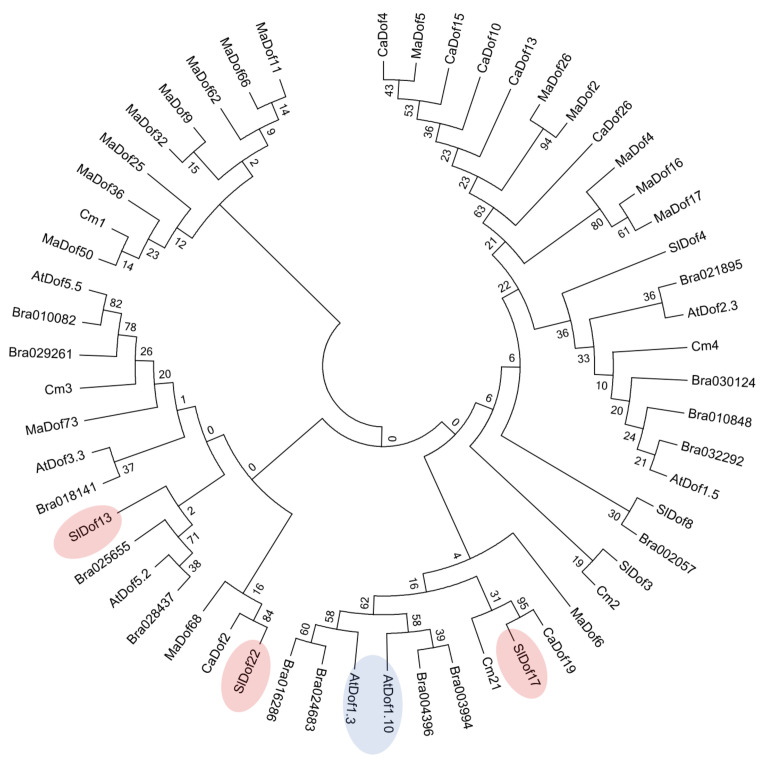
Evolutionary relationships of closest AtzDof1.3 transcription factors based on amino acid sequence. The evolutionary history of the AtzDof1.3 transcription D1 factor sequences was inferred using the Neighbor-Joining method. The bootstrap consensus tree was inferred from 1000 replicates. Evolutionary distances were computed using the Poisson correction method. Evolutionary analyses were conducted in MEGA7. The *A. thaliana* gene loci used to identify the predicted amino acid of homologues in other species included: *AtzDof1.3* or CDF6 (AT1G26790.1); *AtzDof1.10* (At1g69570); *AtzDof5.5* (At5g62430); *AtzDof3.3* or CDF3 (At3g47500); *AtzDof5.2* (At5g39660); *AtzDof2.3* (At2g34140); *AtzDof1.5* (At1g29160). Species names of related zDof TFs are as follows: pepper (*Capsicum annuum*, Ca); chrysanthemum (*Chrysanthemum morifolium*, Cm); banana (*Musa acuminate*, Ma); Arabidopsis (*Arabidopsis thaliana,* At); mustard greens (*Brassica juncea*, Bra); and tomato (*Solanum lycopersicum*, Sl).

To better understand the evolution of *AtzDof1.3* in relation to other *zDofs*, including those in *A. thaliana*, and particularly, those from tomato, the closest homologues of *AtzDof1.3* from *A. thaliana* and other dicots were identified by BLAST, and a phylogenetic tree was constructed (Figure 10). Moreno-Ruiz previously reported that *AtzDof1.3* has a very close paralogue *AtzDof1.10*, which likely resulted from a recent duplication [74]. This close paralogue was also evident in *Brassica juncea*, a member of the mustard family of which *A. thaliana* is also a member, but was not found in the other dicots examined (Figure 10).

### 2.9. Sequence Analysis of AtzDof1.3 Homologues in Tomato

The phylogenetic tree suggested that *SlDof17* (Solyc05g007880.2) is the most likely *AtDof1.3* orthologue in tomato due to the close proximity of branching (Figure 10) and this was confirmed by BLAST of The Arabidopsis Information Resource (TAIR) (accessed 13 September 2022). However, the predicted protein sequence of Solyc05g007880.2, (*SlDof17*) is only distantly related to *AtzDof1.3* [75]. *SlDof17* has many potential protein–protein interactors with players involved in nitrogen metabolism using the STRING database [76]. Of the ten interactors identified, eight were products of characterized genes and five of these proteins, i.e., nitrogen regulatory protein P-II homologue, a nitrogen-sensing protein PII-like gene, glutamate synthase 1, ferrodoxin-dependent glutamate synthase 1 and urease-like all have roles in nitrogen use.

We attempted to clone Solyc05g007880.2 from wild tomatoes species, *S. habrochaites*, *S. pimpinellifolium* and *S. chilense* to determine whether there was significant divergence due to human selection. In our hands, we could only successfully amplify products from *S. pimpinellifolium*. This sequence was almost identical to that published for *S. lycopersicum* (99.7%).

### 2.10. The Role of AtzDof1.3 in Arabidopsis

It was also of interest to determine whether suppression of the *AtzDof1.3* gene in its native *A. thaliana* would lead to alterations in C- or N-phenotypes. We identified an *A. thaliana* T-DNA knockdown of *AtzDof1.3* and determined growth and productivity. The mutants had the same N- and C-content of the control lines. A lower percentage of the mutant seedlings survived at harvest (3-fold lower), and mature plants accumulated less above-ground biomass (2-fold lower) compared to the wild-type. Like the tomato fruit, only shoot fresh weight, not dry weight, was altered. There were no other significant changes in plant growth and development (Supplementary Figure S8). This agrees with recently published work that identifies a role for *AtzDof1.3* in germination of *A. thaliana* [45].

## 3. Discussion

Our hypothesis was that genetically modified lines with deviations in leaf carbohydrates could also have altered source–sink allocation and, hence, growth characteristics [24,44,77]. Sucrose is the primary photoassimilate used for carbon and energy in tomato [78], and our aim was to examine how plants would integrate low sucrose with a general reconfiguration of growth [44]. The line was transformed with *a DNA-binding one zinc finger protein* isoform 1.3 from *A. thaliana*. Any physiological changes associated with this line could be due to a combination of the ectopically expressed TF, positional effects, and somaclonal variation introduced during tissue culture. Still, it was of interest to understand plant plasticity in relation to altered source capacity. Therefore, our first aim was to look at these physiological changes in the transgenic line at multiple levels. Some of the novel traits identified warranted a deeper investigation of *AtzDof1.3*. Thus, our second aim was to perform sequence analysis of the *AtzDof1.3* gene, determine its role in *Arabidopsis* and identify potential homologues in tomato.

### 3.1. L4080 Was Most Likely Altered in Source–Sink Relations

Changes in the relative proportion of starch-to-sucrose in leaf tissue can act as a proxy for modulations in carbon and energy utilization of some plants [24]. Source leaves of L4080 had lower leaf sucrose but normal starch throughout the diurnal period compared to the control (Figure 1) [78]. The causes of lower leaf sucrose in L4080 could be multifaceted: higher respiratory activity exhausting the sucrose pool, lower sucrose synthesis, greater sucrose export from source to sinks, or a combination of these factors [24,78], and none could be explained by our in vitro ^14^C-labeling. While there are no drastic shifts of carbon into the major leaf biomolecular pools, i.e., sugars, starch, protein, organic acids, etc., indicated by ^14^C-labeling, leaf metabolites assayed by GC-MS-TOF in source leaves were altered and, at least in seedlings, the N-to-C ratio changed (Figure 2 and Figure S3, Table 1). The latter indicating co-regulation of the major nutrient pathways in the transgenic line.

In contrast to leaf, there were some differences in ^14^C-partitioning in the sugar fraction of the pericarp during fruit development, sometimes incongruent with steady-state levels (Figure 1E,F and Figure 2A,B). These changes in carbohydrate pools did not ramify to other pathways as fruit metabolites were fairly constant in L4080 compared to the control (Figure 7C,D; Supplementary Table S1). One interesting correlation was between higher fruit sucrose (Figure 1F), increased fruit size, and fruit mass (Figure 3) at 49 DPA, which led to higher yields. Higher solids could drive increased fruit import of water, explaining higher fruit expansion and mass at this stage [79]. This higher mass decreased as the fruit developed (Figure 3). We observed that L4080 showed greater signs of fruit cracking, which could accelerate evapo-transpiratory loss, and lower fruit mass, but this was not enough to increase total sugars or brix in the ripe fruit (Figure S3).

### 3.2. L4080 Varied in N-Use and Root Growth in Juvenile Plants

The transgenic line studied here might have novel mechanisms for N-use and root growth in 6-week-old plants (Figure 4, Figures S4 and S5). When plants were cultured in vermiculite, there was evidence for enhanced root foraging in L4080 and, better above- and below-ground biomass compared to the control genotype in response to lower N (Figure 4). L4080 dry matter was lower than the control line under normal N in this experiment (Figure 4).

Nitrogen and carbon metabolism are inextricably linked, and potential co-regulation of these pathways was illustrated when plants were grown at higher CO_2_. L4080 was able to increase in biomass at 760 ppm CO_2,_ which was not evident in the wild-type (Figure 5). This, plus the response to low N, supports the idea that this L4080 has better N-utilization compared to the control. N-limitation often inhibits a plant’s ability to scavenge additional carbon [62,80]. We propose that the high endogenous N in L4080 may have played a role in relieving that inhibition (Supplementary Figure S4) when plants were ‘fertilized’ with carbon (Figure 5). This conclusion is hampered by the lack of critical data on N-species, i.e., NO_2_^−^, NO_3_^−^ or NH_4_^+^ in the plant tissue as only total N was assayed [81].

### 3.3. Post-transcriptional Modification May Be an Important Driver in The Phenotypic Changes in L4080

Only 0.39% of the 9245 cDNAs on the Affymetrix array changed in expression (*p* < 0.01; Supplementary Table S2). This was surprising since additional copies of a TF may be expected to alter the expression of multiple target genes, and that changes to these primary target genes could have pleiotropic effects on others. Further, transcripts of genes involved in N- and C-regulation did not vary (*p* < 0.01) in L4080 as shown in other reports where *zDof* overexpression altered metabolism [30,36]. Transcripts derived from genes with zDof *cis-elements* were disproportionately upregulated (Supplementary Table S3), indicating that the *AtzDof1.3* transcript, which was detected by semi-quantitative RT-PCR in the source leaves used for transcriptomics (Supplementary Figure S1) was likely active. We offer a few explanations for these observations: (a) Changes in phenotype may have been brought about by waves of expression of key transcription factors over time that were not captured by our single transcriptomic analysis. *AtzDof1.3* may have targeted downstream transcription factors (Figure 8). Thus, there may be incremental but broad changes in gene expression. (b) Post-transcriptional changes may have been more important in explaining changes in phenotype than changes in transcript.

In contrast to the gene expression data, more dynamism was detected in the metabolite profiles of the equivalent leaf, which suggests that post-transcriptional activity was more pronounced, or easier to detect at that time point (Figure 6 and Figure 7) in L4080. (c) The experimental design used may have acted as a source of variability thus reducing the number of significant differences between genotypes. An individual leaf was sampled from a population of over 100 plants grown in a randomized complete block design in a greenhouse so that each sample used for RNA extraction was harvested from a distinct microenvironment. Our design better mimics real-world commercial conditions, and differences between the control and L4080 may be more reproducible long-term. Still, RNA-Seq transcriptomics may reveal more widespread changes of greater intensity in L4080 than possible with the Affymetrix array. Collectively, these data support recent views that some transgenic lines with detectable changes in agronomic traits may not always vary substantially in their transcriptome from the control lines [82,83].

### 3.4. The Evolutionary History of AtzDof1.3 and Homologues in Tomato Differ

If an assumption is made that *AtzDof1.3* is partially responsible for the phenotypes seen here, then a logical follow-on question would be to determine if there is a close orthologue of *AtzDof1.3* native to tomato, and whether over-expression in tomato would lead to a recapitulation of some of the traits observed here. To address the first question, a phylogenetic tree of *AtDof1.3* and its closest paralogues were analyzed, and it indicated that there is most likely no true orthologue of *AtzDof1.3* in tomato because there is a duplicated gene in Arabidopsis (*AtDof1.10*) that may have undergone sub- or neofunctionalization [74]. This may complicate efforts to easily elevate levels of a near-identical protein in tomato using conventional genetic engineering approaches.

Further, we could only amplify *SlDof17* from the closest tomato relative, *S. pimpinellifolium*, and not *S. habrochaites* and *S. chilense*, although multiple primers were used (Supplementary Figure S7). It is possible that there was minimum conservation at the sequence used to design the primer in *S. lycopersicum* and the orthologous sites in *S. habrochaites* and *S. chilense*. The development of high-quality whole genome sequences of these wild species would help to address this question.

### 3.5. AtzDof1.3 May Be Important for Germination of Arabidopsis

If ectopic expression of *AtzDof1.3* in tomato was partially responsible for altered N- and C-use then it may be possible to identify such changes in T-DNA knockouts of the gene in *Arabidopsis*. However, there were no detectable changes in C- and N-levels in the *Arabidopsis* T-DNA knockouts. In maize, reduced *zDof1* in a *RescueMu* transposon-tagged line also did not change metabolism in that species [58]. Functional redundancy among the *zDof* family may explain these observations. In *Arabidopsis*, the presence of the duplicated *AtzDof1.10* may have compensated for loss of *AtzDof1.3*. Disruption of the *AtDof1.3* gene, however, may have reduced germination capacity and led to differences in water relations, as aboveground fresh weight was lower (Supplementary Figure S8). Digital gene expression data show that *AtDof1.3* (TAIR locus AT1G26790.1) is activated under cold and osmotic stress conditions [73]. Exposing the T-DNA knockouts to mild cold may have led to an exacerbated phenotype.

### 3.6. Summary

We showed that a transgenic tomato line ectopically expressing *AtzDof1.3* had 25% higher yield in a greenhouse at 49 DPA, due to 25% higher fruit mass. This correlated with higher fruit sucrose at this developmental stage, which could increase osmotically driven water import. In young plantlets, L4080 was able to convert higher CO_2_ levels into increased biomass, and grew better in another experiment on 1/10 reduced N compared to the wild-type, facilitated in part by greater root elongation. In fruit at 14 DPA, ^14^C-feeding to pericarp disks also suggested greater flux towards amino acids, organic acids, and respiration and away from sugars and starch. GC-MS-TOF profiling indicated that most changes in metabolite levels were in the leaf amino acid pool. Our analysis was conducted on a single genotype, however, transient expression of *AtzDof1.3* in tomato tissues, recapitulated the altered carbohydrate metabolism found in L4080. Collectively, these data are consistent with altered N- and C-metabolism of L4080. This work suggests that a broader study of this gene may be warranted as the traits associated with this line may be useful in agricultural applications.

## 4. Materials and Methods

### 4.1. Generation of Transgenic Lines

The transgenic lines were created by Mendel Biotechnology Inc., (Hayward, CA, USA), and Seminis Vegetable Seeds (Monsanto, Woodland, CA, USA). The tomato genotype used as the parental line was *S. lycopersicum* cv. ‘T63’ a ‘semi-determinate’ beefsteak variety. To create the tomato plants ectopically expressing the *PD::AtzDof1.3*, two separate constructs; an activator construct containing the phytoene desaturase promoter, and a target construct containing the *AtzDof1.3* transcription factor were transformed separately into individual plants. These two lines were crossed and T_1_ plants inheriting both constructs were selected [84,85]. The method used to create these lines was described in detail in Powell et al. [86]. All characterization was performed on T_4_ and subsequent generations, each time checking for the presence of the constructs by PCR.

### 4.2. Growth Conditions

Most experiments were conducted on plants grown under greenhouse conditions from April to August 2008 as described [87]. The initial characterization took place in Summer 2006 on unpruned plants. Thereafter, the plants were pruned to two fruit per inflorescence and determinate growth was promoted by topping the plants after the 9th inflorescence. The *PD::zAtDof1.3* line referred to as L4080 and the non-transformed control were analyzed. An ‘empty vector’ line containing the activator and target construct only were included initially in analyses assaying biochemical parameters. However, they did not differ from the untransformed control and were therefore not included in subsequent experiments.

*Growth under nitrogen limitation.* Seedlings were transplanted into 4-inch pots in a soilless growth media using vermiculite as a solid support, with each pot containing both the control and the transgenic line. One-third of the plants were supplied with 50 mL of ½ strength Hoagland solution each day, and the remaining were treated with either 1/10 and 1/20-fold less nitrogen (N) adjusted to give equal osmolarity by replacing Ca(NO_3_)_2_ and KSO_4_ with CaCl_2_ and KSO_4_ [88]. Samples were harvested after 6 weeks of growth and assayed for the length, fresh and dry weight and carbon and nitrogen content of the above and below ground tissue.

*Growth under elevated carbon dioxide.* Seedlings were transplanted into 6-inch pots and grown in the greenhouse for two weeks. Pots containing plants that grew uniformly were transferred to a Controlled Environment Facility into two different chambers, one with 360 ppm and the other with 720 ppm CO_2_ and grown for 6 weeks. Light supplementation was 12 h:12 h day length with metal halide lamps. The plants were supplemented with ½ strength Hoagland solution when transferred to the growth chambers, i.e., after two weeks of growth in the greenhouse. The aboveground component was harvested after 4 weeks of growth in the chamber. Fresh, and fresh and dry weights were determined.

### 4.3. Detection of The AtzDof1.3 Transgene in Tomato

DNA was extracted as described by Leterrier et al. (2008) [89]. The *A. thaliana* transgene was amplified using primers designed to the construct borders flanking the site of *AtzDof1.3* transgene *LexA*: 5′-TACAGTACGTCGAGGGGATGAT-3′ and E9 terminator sequence 5-CTGGTGTGTGCGCAATGAAACTG-3′ of the target vector. The PCR reaction (25 μL) contained 40 ng of DNA, 2× Advantage GC-Melt Buffer, dNTP, LA Polymerase (Clontech, Palo Alto, CA, USA), and 0.4 μM primers. The PCR conditions were as follows: an initial start at 94 °C for 3 min, 30 × cycles of denaturation at 94 °C for 15 s, annealing at 60 °C for 1 min, an extension at 68 °C for 1 min, and a final extension at 68 °C for 5 min. DNA was subcloned into TOPO XL (Invitrogen, Valencia, CA, USA). The nucleotide composition of these fragments was determined using ABI BigDye Terminator sequencing. Gene identification was performed by querying GenBank and the Sol Genomics Network database using the Basic Local Alignment Sequence Tool (BLAST) [90]. The promoter sequence was identified using primers designed to the *LexA* gene 5′-GCCTTCAGATGTTCTTCAGC-3′ as the antisense primer, and *phytoene desaturase* gene 5′-TAACTGCCAAACCACCACAA-3′ as the sense primer. The PCR reaction was performed using AmpliTaq DNA Polymerase (Applied Biosystems, Waltham, MA, USA) following the protocol exactly as described.

### 4.4. Semi-Quantitative Reverse Transcription Polymerase Chain Reaction (Semi-qRT-PCR)

RNA was extracted from various plant tissues: developing fruit at 7, 49 (mature green) and 63 (red) days post-anthesis (DPA); source and sink leaves; and flowers using Trizol [89] and treated with DNAse I (Promega, Madison, WC, USA). A total of 700 ng RNA was used for a duplex reverse transcription using the protocol described in [89], except for two antisense primers that were used for cDNA synthesis including the Actin housekeeping gene 5′-GCAGCTTCCATTCCAATCAT-3′ and the *AtzDof1.3* gene specific primer 5′-CCGGTTAAATTGTTTGGCTGGA-3′. The PCR reaction (25 μL) contained 4 μL of cDNA, 4 primers; the antisense primers for both genes as well as the Actin sense primer 5′-CAGGGACGTGAAAGAAAAGC-3′ and that for the *AtzDof1.3* 5′-AGGGGATGCTTGGGGAGGAAGT-3′ at 0.1 μM. The PCR conditions were as follows: an initial start at 94 °C for 1 min; 30 × cycles of denaturation at 94 °C for 30 s, annealing at 60 °C for 30 s, and extension at 72 °C for 2 min; and a final extension at 72 °C for 10 min. Three separate amplifications were performed on tissues harvested from three different plants. The intensity of the amplified bands was determined using the imaging software equipped within the AlphaImager 3400 (Alpha Innotec; Santa Clara, CA, USA) molecular imaging system.

### 4.5. Eco-Physiology and Biochemical Measurements

A total of 108 plants of both genotypes, i.e., L4080 and the control line, were greenhouse-grown from April to August 2008 in a randomized block design to ensure adequate replication. Leaf and fruit physiological analyses were performed at 7-day intervals after anthesis, i.e., 7, 14, 21, 28, 35, 42 DPA, breaker (49 DPA) and red ripe fruit (~63 DPA). Parameters indicating growth included the following: (i) internode growth, which was assessed by weekly measurements of the length of the fourth truss from 21 plants per genotype until it remained constant, and (ii) leaf growth, which was determined by assaying the length of the leaves located above the fourth truss. To assess yield, plants that did not undergo fruit pruning were used. Plants were pruned to only allow the growth of nine trusses. Flower and fruit number per plant, fruit fresh weight, and fruit circumference were taken each week. Yield and shoot and root dry weight measurements were at the end of the experiment. Fruits were removed, counted, and weighed. The remaining vegetative tissue was divided into shoot (above ground) and root (below ground), and the materials were dried at 55 °C for three weeks to determine dry weight. Yield was calculated as (fruit fresh mass at breaker (g) × fruit number).

Analyses of fruit and leaf carbohydrate content and ^14^C-partitioning were conducted also as previously described [50]. Chlorophyll was assayed using acetone [91], and Total C and N was determined by combustion analysis at the UC Davis DANR Analytical facility [92]. For carbohydrate and GC-MS-TOF metabolite analysis of leaf and fruit (see below), one fruit and the adjacent leaf were harvested from the same plant between 12 to 1 PM. A total of six biological replicates were used, where one replicate represents a single plant and only one plant was sampled per developmental stage.

### 4.6. GC-MS-TOF Metabolite Profiling

For the fruit, the pericarp was sampled from the fruit equator, while the whole leaf was sampled as described in [50]. Tissue was frozen in liquid nitrogen, homogenized, and then treated as described by [93]. GC-MS-TOF was performed at the Australian Centre for Plant Functional Genomics, Melbourne as described in [63]. Multivariate analysis including Partial Least Squares Discriminant Analysis (PLS-DA) and heat map generation was performed using Metaboanalyst (TMIC, Edmonton, AB, Canada) as outlined in [94].

### 4.7. Analysis Using the Affymetrix GeneChip

Leaves adjacent to fruit at 21 DPA were harvested from six individual plants at midday. RNA was extracted using Trizol [89], and the integrity of the resultant RNA was checked using the Agilent 2100 BioAnalyzer system (Agilent, Waldbronn, Germany). For each genotype, six pools of RNA were isolated, and two of each were combined to produce three biological replicates. Gene expression, hybridization, and basic analysis were performed at the UC Davis Medical School, Sacramento, CA, USA [95]. RNA was hybridized using the Affymetrix GeneChip Tomato Genome Array. The data underwent RMA normalization followed by a baseline transformation using GeneSpring GX 11.5 (Agilent Technologies, Santa Clara, CA, USA) and dChip [96]. Differentially expressed genes (DEGs) were identified at *p* < 0.01. Affymetrix cDNAs were analyzed by BLAST against GenBank and gene IDs based on the tomato genome v. ITAG 2.4 were extracted.

### 4.8. A. thaliana zDof1.3 Construct Assembly and Transient Expression

Primers were designed to AT1G26790 using the online software Primer3 (v. 0.4.0, http://bioinfo.ut.ee/primer3-0.4.0/ accessed on 20 December 2018) and were checked for specificity in NCBI Primer Blast (https://www.ncbi.nlm.nih.gov/tools/primer-blast/; accessed on 20 December 2018). The forward primer was (5′-GGACTCTTGACCATGCTCTTATACATACTCTTGTCTCAGA-3′), and the reverse primer was (5′-GTCAGATCTACCATGTTATATGCTCTCTCTGAAGTTCA-3′), covering the longest open reading frame (ORF) region. The full length of *zDof1.3* was amplified from *A. thaliana* genomic DNA by Phusion polymerase (NEB,) using the PCR reaction (35 cycles at 98 °C for 30 s, 60 °C for 20 s, and 72 °C for 50 s). PCR amplicons were purified from a 1% (*w*/*v*) agarose gel using a QIAquick^®^ Gel Extraction Kit (QIAGEN, Hilden, Germany). Plasmid pCAMBIA1304 was linearized by the restriction enzyme *Nco*I, and *AtzDof1.3* was fused into pCAMBIA1304 through In-Fusion^®^ cloning (Takara Bio, Kusatsu, Japan). The assembled construct was transformed into *E. coli* chemically competent cells (strain ‘DH5-alpha’). After 12–16 h incubation, the collected cell culture was used for the isolation of the plasmid using the QIAprep^®^ Spin Miniprep Kit (QIAGEN, Hilden, Germany). The purified plasmid was sequenced for the presence of an unmutated *AtzDof1.3* fragment.

*Transient expression of AtzDof1.3 in tomato seedlings.* The purified plasmid constructs were transformed into electrocompetent *A. tumefaciens* strain LBA4404 by electroporation. Transformed *A. tumefaciens* were kept at −80 °C in 50% (*v*/*v*) glycerol. The plant transformation procedure was achieved using the FAST method (Li and Nebenführ, 2010). Briefly, 4-day-old tomato seedlings (*S. lycopersicum* L.) were cocultured with the *A. tumefaciens* cell resuspension under dark for 60 h. At three-days post-infection, seedlings were selected for GFP-positive signal.

*RNA isolation and semi-quantitative RT-PCR.* Total RNA was extracted from GFP-positive seedlings 5-days post-infection (at least three biological replicates for each genotype) using a modified TRIzol method (Wang et al., 2008). In brief, 100 mg of seedlings were ground in liquid nitrogen and homogenized in 1000 µL of TRIzol buffer (38% (*v*/*v*) phenol adjusted pH to 4.0 with Tris-HCl buffer, 0.8 M guanidine thiocyanate, 0.4 M ammonium thiocyanate, 0.1 M sodium acetate (pH 5.0), and 5% (*v*/*v*) glycerol) by vortexing. The upper aqueous phase from the centrifuged (4 °C, 16,000 rpm) homogenate was transferred into a new tube and mixed with 500 µL isopropanol followed by 10 min centrifugation at 4 °C. The collected pellet was treated with 8 M LiCl overnight, then treated using Promega™ RQ1 RNase-Free DNase (Thermo Fisher Scientific, Waltham, MA, USA) according to the manufacturer’s instructions. Another 50 µL LiCl was added and incubated at −20 °C for 60 min. After centrifugation, the pellet was air-dried, washed with 70% (*v*/*v*) ethanol, and resuspended in 20 µL RNase-free water.

A High-Capacity cDNA Reverse Transcription Kit (Thermo Fisher Scientific, Waltham, MA, USA) was used to synthesize the first-strand cDNA from total RNA. Briefly, 10 µL RNA (2 µg) was mixed with 2 µL 10× RT Buffer, 0.8 µL dNTP Mix (100 mM), 2 µL RT Random Primers (10×), 1 µL MultiScribe™ Reverse Transcriptase, and 4.2 µL Nuclease-free H_2_O. The thermal cycler was set at 25 °C for 10 min, 37 °C for 120 min, 85 °C for 5 min, and 4 °C hold.

PCR was performed using a specific primer pair of the *AtzDof1.3* transcripts: the forward primer was Dof1.3_semiq_F, 5′-CGACCACACATGATGAATAACC-3′, and the reverse primer was Dof1.3_semiq_R, 5′-GCTGGTTAACGTTGTAGTTGTT-3′. *Actin* was used as the control gene, with the upstream primer (5′-GCTATCCAGGCTGTGCTTTC-3′) and the downstream primer (5′-CAGTAAGGTCACGACCAGCA-3′). PCR program was optimized with 25 cycles at 95 °C for 15 s, 53 °C for 30 s, and 72 °C for 20 s using AmpliTaq™ DNA Polymerase (Thermo Fisher Scientific, Waltham, MA, USA).

Seedling samples were cleaned with water and stained with Lugol’s iodine solution (5 g KI and 0.5 g I_2_ in 500 mL water) for 30 min, then de-stained using water until color difference was observed.

### 4.9. Analysis of the Predicted Amino Acid Sequence of AtDof1.3 and Its Homologues

The evolutionary history of the *AtzDof 1.3* and the closest homologues were inferred using the Neighbor-Joining method [97]. The bootstrap consensus tree inferred from 1000 replicates is taken to represent the evolutionary history of the zDof sequences analyzed [98]. Branches corresponding to partitions reproduced in less than 50% bootstrap replicates were collapsed. The evolutionary distances were computed using the Poisson correction method [99] and are represented in units of the number of amino acid substitutions per site. There were 56 amino acid sequences including seven predicted from the following *A. thaliana* gene loci: *AtzDof1.3* (AT1G26790.1); *AtzDof1.10* (At1g69570); *AtzDof5.5* (At5g62430); *AtzDof3.3* (At3g47500); *AtzDof5.2* (At5g39660); *AtzDof2.3* (At2g34140); *AtzDof1.5* (At1g29160), and related zDof TFs from pepper (*Capsicum annuum*, Ca); chrysanthemum (*Chrysanthemum morifolium*, Cm); banana (*Musa acuminate*, Ma); Arabidopsis (*Arabidopsis thaliana*, At); mustard greens (*Brassica juncea*, Bra); and tomato (*S. lycopersicum*, Sl). All positions containing gaps and missing data were eliminated. There were 46 positions in the final dataset. Evolutionary analyses were conducted in MEGA7 [100].

### 4.10. Analysis of AtDof1.3 in Arabidopsis Using T-DNA Knockouts Lines

An *A. thaliana* T-DNA insert mutant for *AtDof1.3* (AT1g26790) was procured from The Arabidopsis Biological Resource Center [101]. The *AtDof1.3* mutant line, CS435276, and the control, CS60000, were selfed for three generations, and homozygotes were selected via PCR. Oligonucleotide primer sequences specific to the *AtDof1.3* locus, 5′-ATATTGACCATCATACTCATTGC-3′ and 5′-GAAACAGGTTCAGCGTTTATCTTC-3′, were provided by GABI-KAT [102,103]. In the laboratory, plants were grown in 6-inch pots containing UC soil mix under long day conditions (16-h-light/8-h-dark cycle) at 22 ± 1 °C.

### 4.11. Statistical and Network Analysis

All plants were grown in a randomized block design. A one-way ANOVA at *p* < 0.05 was used to detect significant changes among samples using SAS Statistical Software (SAS Institute Inc. 2010; Cary, NC, USA); however, for microarray data, differences were deemed significant at *p* < 0.01. Prior to multivariate analysis, metabolomics data were log_10_ transformed to approximate to a normal distribution.

To develop a transcriptional network using Affymetrix data, the region 2000 bp upstream of the translation start site of each gene was scanned for the *zDof cis*-elements ([A/T]AAAG) as described by [104,105,106], using the Find Individual Motif Occurrence (FIMO) software in Motif-based sequence analysis tools (MEME suite) [107].

## Figures and Tables

**Table 1:** Leaf metabolites differing in content between the control and L4080. The table shows relative values i.e., the fold difference of that metabolite in L4080 relative to the control when harvested when adjacent fruit was 21 DPA or Breaker. Only the emboldened values differed at significance level *p* < 0.05. Metabolites classified as, or chemically related to amino acids are italicized.

Metabolites	L4080 vs. WT			
	21 DPA		Breaker	
	x-fold	*t*-test	x-fold	*t*-test
***Aspartate***	**0.818**	**0.005**	**3.841**	**0.002**
Citrate	**0.696**	**0.014**	1.093	0.179
Fucose_MX2	2.556	0.706	**1.832**	**0.039**
Galactonate	**1.155**	**0.000**	1.165	0.176
Galacturonate_MX1	**83.124**	**0.002**	3.627	0.052
Gluconate	**1.255**	**0.023**	1.189	0.257
Glucose_MX2	2.069	0.215	**3.219**	**0.020**
Glucose-6-p_MX1	0.806	0.298	**2.029**	**0.011**
Glucose-6-p-MX2	0.913	0.158	**1.775**	**0.04**
Glucuronate_MX1	**3.589**	**0.003**	1.713	0.456
***Glutamate***	0.599	0.799	**2.157**	**0.005**
Heptanoic acid_1TMS	**0.540**	**0.008**	0.926	0.929
Inositol	0.901	0.603	**0.736**	**0.046**
Lactic acid	**0.739**	**0.035**	1.042	0.897
Maltose_MX1	**1.350**	**0.002**	0.838	0.833
Mannitol	0.840	0.238	1.061	0.883
Melibiose_MX1	0.530	0.176	**3.198**	**0.012**
Oxalate	**0.459**	**0.027**	1.494	0.063
***Phenylalanine***	**1.934**	**0.000**	**1.828**	**0.021**
***Pyroglutamate***	0.630	0.491	**1.651**	**0.000**
Ribonate	**0.853**	**0.008**	**1.829**	**0.009**
Saccharic acid	**0.869**	**0.000**	1.140	0.524
***Shikimate***	**1.212**	**0.002**	**1.618**	**0.007**
Threonate-1,4-lactone	**0.092**	**0.000**	0.584	0.189
***Threonine*** 3TMS	**0.685**	**0.028**	**3.307**	**0.001**
***Tyramine*** 3TMS	**1.270**	**0.000**	**1.835**	**0.016**
***Tyrosine***	1.043	0.117	**0.267**	**0.016**
Xylose_MX2	**2.946**	**0.004**	1.940	0.224
Unknown	**0.502**	**0.045**	1.587	0.070
Unknown	**0.521**	**0.005**	0.866	0.489
Unknown	0.550	0.427	**2.812**	**0.003**
Unknown	0.585	0.311	**2.707**	**0.001**
Unknown	**0.632**	**0.038**	3.259	0.181
Unknown	0.638	0.095	2.164	0.051
Unknown	0.695	0.090	**2.168**	**0.022**
Unknown	0.784	0.133	**1.643**	**0.001**
Unknown	0.831	0.879	**1.911**	**0.040**
Unknown	**0.837**	**0.049**	**1.772**	**0.021**
Unknown	0.848	0.060	**2.279**	**0.000**
Unknown	**0.948**	**0.000**	**1.478**	**0.002**
Unknown	0.962	0.16	**1.681**	**0.043**
Unknown	**0.986**	**0.003**	**1.295**	**0.010**
Unknown	1.180	0.078	1.514	0.100
Unknown	**1.447**	**0.003**	1.223	0.173
Unknown	**1.518**	**0.006**	0.597	0.265
Unknown	**1.605**	**0.007**	0.838	0.833
Unknown	**1.967**	**0.015**	1.522	0.123
Unknown	**2.129**	**0.007**	1.849	0.785
Unknown	**2.788**	**0.027**	0.943	0.737
Unknown	**3.318**	**0.021**	0.190	0.065

## Data Availability

All data available upon request.

## Supplementary Materials

The following supporting information can be downloaded at: https://www.mdpi.com/article/10.3390/ijms231911229/s1. References [108,109,110] are cited in the Supplementary Materials.
