# Supplementary material for: Ectopic Expression of Arabidopsis thaliana zDof1.3 in Tomato (Solanum lycopersicum L.) Is Associated with Improved Greenhouse Productivity and Enhanced Carbon and Nitrogen Use
Source: Int J Mol Sci. 2022 Sep 23;23(19):11229. doi: 10.3390/ijms231911229 (PMC9570051; doi:10.3390/ijms231911229)
Supplement: Supplementary file 1 [file ijms-23-11229-s001.zip › ijms-1901155-supplementary.pdf]

Supplementary Material

Ectopic expression of *Arabidopsis zDof1.3* in tomato (*Solanum lycopersicum* L.) is associated with improved greenhouse productivity through enhanced carbon and nitrogen use

Kietsuda Luengwilai [1†], Jingwei Yu[1], Randi C. Jiménez,[1,§], Andrea Vega[2], Maysaya Thitisaksakul[1ξ], Shaoyun Dong[1,‡], Diane M. Beckles[1*]

[1]Department of Plant Sciences, University of California, One Shields Avenue, Davis CA 95616

[2]Faculty of Engineering and Sciences, Universidad Adolfo Ibáñez. Millennium Nucleus for the Development of Super Adaptable Plants (MN-SAP). Center of Applied Ecology and Sustainability (CAPES), Santiago, Chile.

.

Figure S1. Semi-quantitative RT-PCR to detect *AtzDof1.3* in L4080 tissues.
Figure S2 Leaf sugar content in control and transgenic tomato line L4080.
Figure S3. Total sugar content in the developing fruit of control and transgenic tomato line L4080.
Figure S4 Total N and the Carbon-to-N ratio in control and L4080 plants grown under varying N.
Figure S5 Root characteristics of tomato control and transgenic Line 4080.
Figure S6 Pictures of mature control and L4080 tomato plants in the greenhouse.
Figure S7 PCR amplification of AtDof1.3 orthologue SlDof17 in wild tomato species.
Figure S8 Physiological comparison of wildtype Arabidopsis and *AtDof1.3* T_4 (T-DNA insert line).
Table S1. Relative levels of metabolites in fruit pericarp sampled at different developmental stages in the control and L4080.
Table S2. Changes in gene expression using the GeneChip® Tomato Genome Array.
Table S3. Identification of the predicted *cis*-elements motifs in promoters of Dof-regulated genes.
Table S4. Members of the Dof gene family with proven roles in altering metabolism and storage product accumulation.

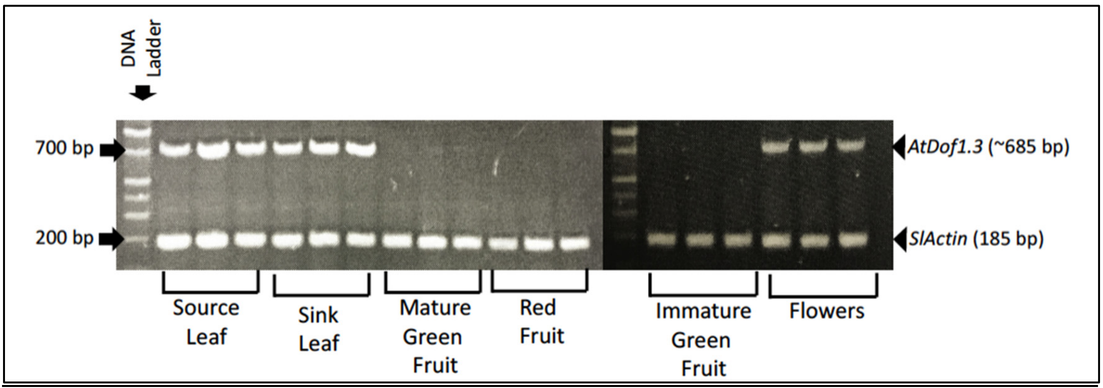

Figure S1. Agarose gel showing the semi-quantitative RT-PCR products of AtDof1.3 and actin amplified from different L4080 tissues. Each RT-PCR reaction consisted of three biological samples derived from RNA harvested from three separate plants. Actin was included in the reaction as a housekeeping control. There was no amplification of AtDof1.3 in the non-transformed control line (data not shown).

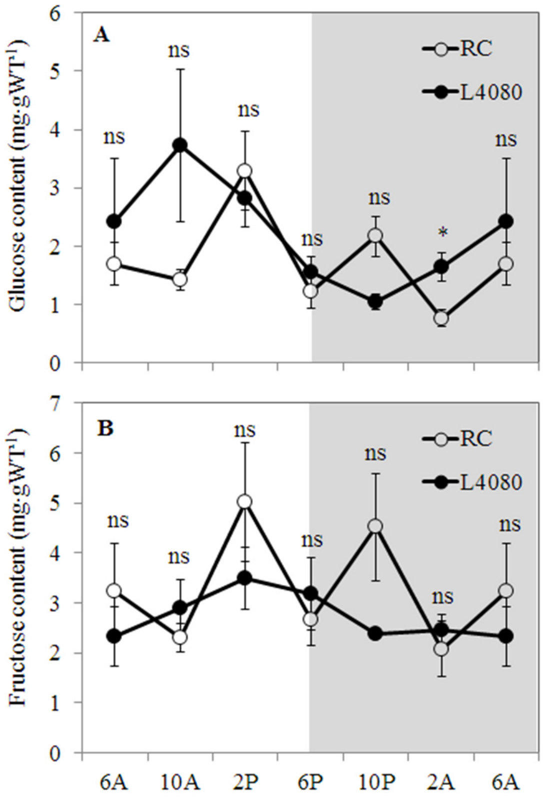

Figure S2. Leaf sugar content in control and transgenic tomato line L4080. Changes in leaf carbohydrate content (g.FWT^{-1}) during the diel, where the shaded area represents the night period. **A**. Glucose, **B**. Fructose. Data is the mean ± SEM of 6 biological replicates. An asterisk indicates data points differing between genotypes (*P*<0.05). ns = not significant. L4080 is the AtzDof1.3 line, and RC- is the untransformed control.

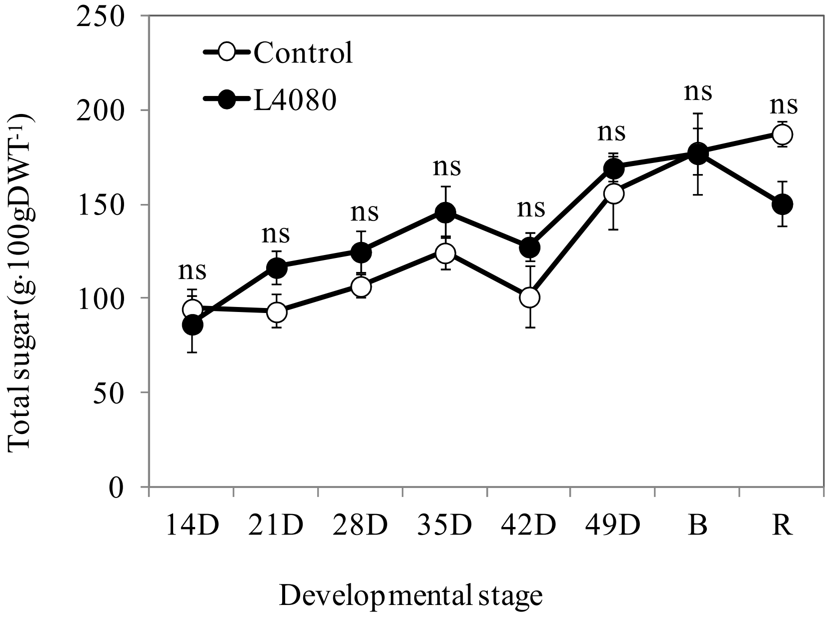

Figure S3. Total sugar content in the developing fruit of control and transgenic tomato line L4080. Fruit carbohydrate assayed in the pericarp taken at 7-day intervals during fruit development. DPA – days post anthesis, B- fruit at Breaker stage ~ 63 DPA and RR red ripe ~70 DPA.

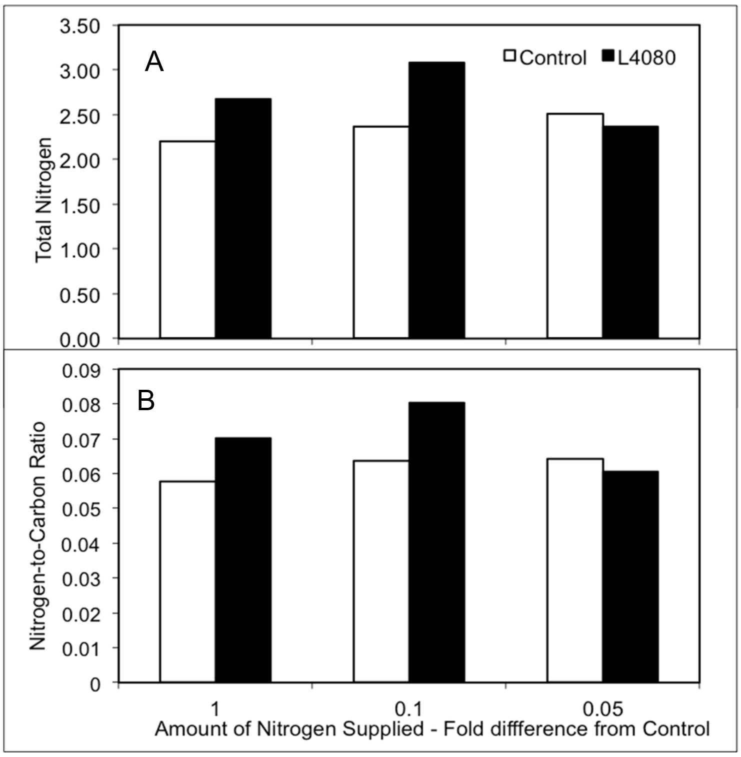

Figure S4. Total N (A) and the Carbon-to-N ratio (B) in control and L4080 plants grown under varying N. Six plants of each genotype, grown under each condition was ground and analyzed. Data is for duplicate samples.

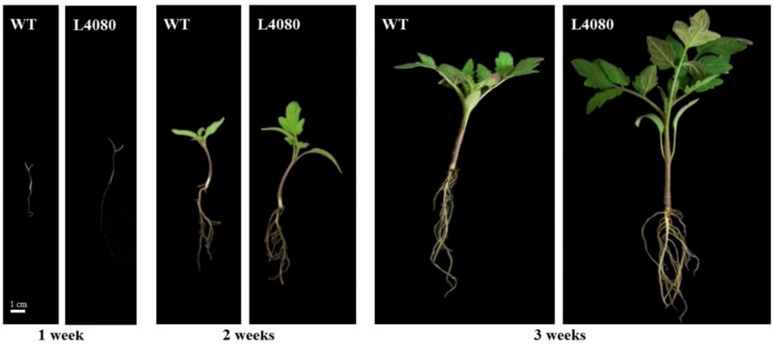

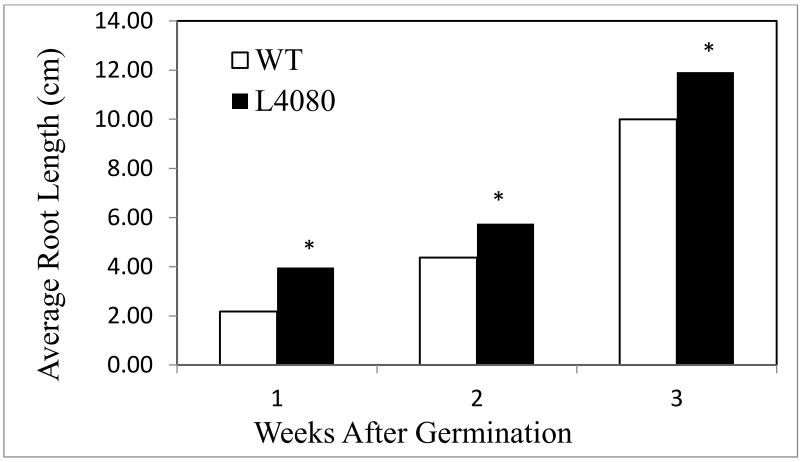

Figure S5. Root characteristics of tomato control and transgenic Line 4080. Six plants of each genotype were used for each week of measurement. Plants were grown in one-gallon pots to promote free growth of roots. The plants were carefully extracted from the soil, washed in deionized water, and blotted dry with paper towels. **A)** Each plant was photographed, and representative plants of each genotype are shown. **B)** Root length was measured by taking a digital image and counting the length of the main lateral root in pixels. This measurement considers that the roots are not straight. An ANOVA was performed on the data using SAS9.1.3 (**Figure 2.3**) (Cary, NC).

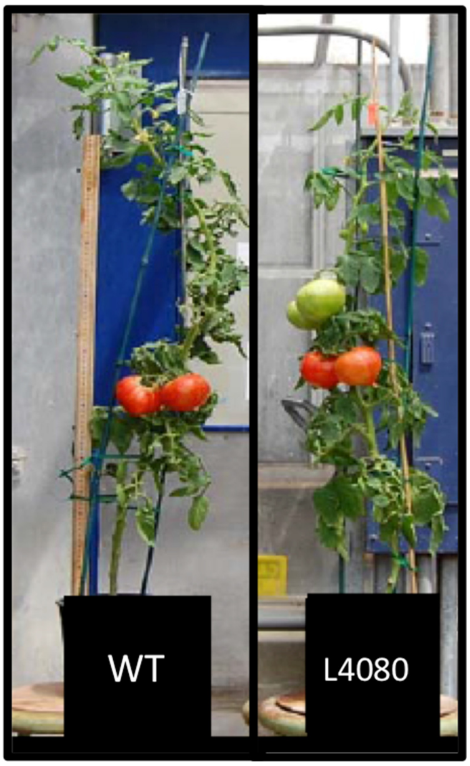

Figure S6. Pictures of mature control and L4080 tomato plants in the greenhouse. Plants were pruned to 2 fruit per inflorescence and terminated after the 9th truss. There was no significant

difference in plant height, leaf area, rate of leaf appearance, number of trusses when the data collected from 12 plants were averaged.

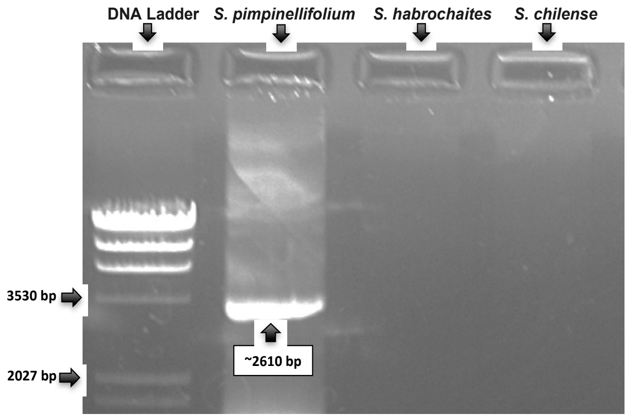

Primer Name	Primer Sequence
s49	CCCATCTTACCCCTTTTTCA
as885	CATCACCAGATCAAACATCAAAG
s1021	TCAGATCGTGAGGGTGTAGC
s1786	AGCAGGTCGAGTTGAAGCAG
as1846	TCCATTTGTCCTTGAAGCAG
s2440	TTCGACTCGTGGTACAATGC
as2830	AAACTTCAGCTGCATCTTATTTATCTC

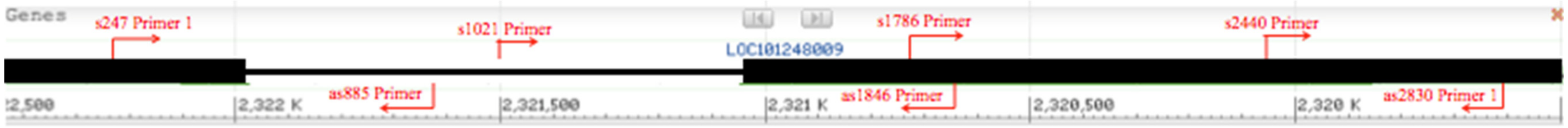

Figure S7. PCR amplification of wild tomato genomic DNA using *SlDof17* primers. **A**. PCR product amplified using primers s247 and as2830. There was no amplification from *S. habrochaites* or *S. chilense.* **B, C**. The sequences and primer location relative to each other on *SlDof17*. These different primer combinations were used for various PCR reaction. In our hands, there was no amplification of an orthologue from the wild species. Genomic DNA was extracted from *S. pimpinellifolium, S. habrochaites, S. chilense,* and *S. lycopersicoides* (not shown). The PCR product was purified using QIAquick gel extraction kit (Qiagen) and ligated into pGEM® T Easy Vector (Promega) for sequencing. *SlDof17* in *S. lycopersicum* (Sequence ID:) were downloaded from NCBI. The multiple sequences alignment was done using the Clustal Omega http://www.ebi.ac.uk/Tools/msa/; (accessed February 25th 2017)**.**

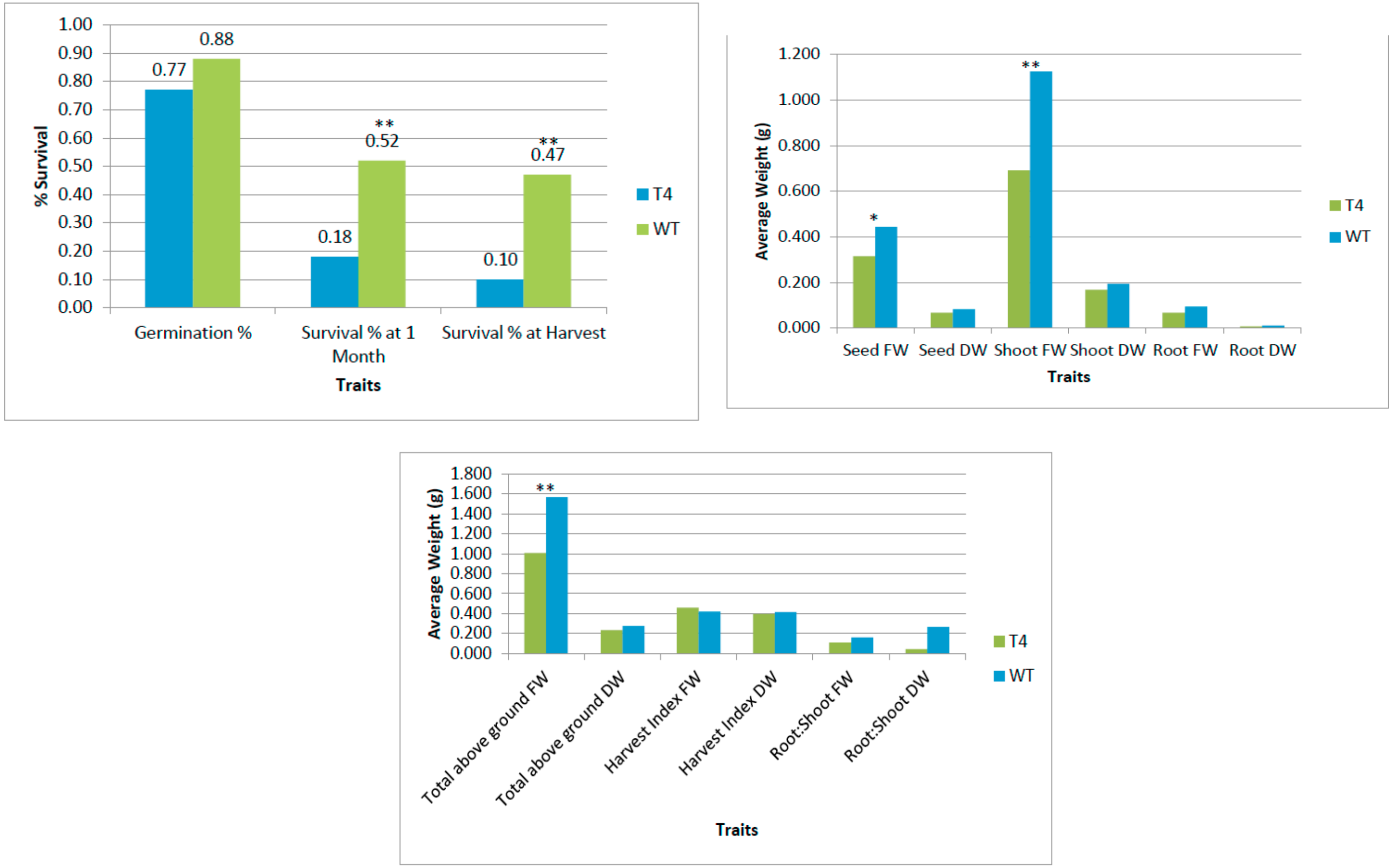

Figure S8. Physiological comparison of wildtype Arabidopsis (WT) and *AtDof1.3* T4 (T-DNA insert line –T4). **A**. Average percentages of germination, survival at one month (32 days) and survival at harvest (61 days) **B**. Analyses of individual plant tissue average weights . **C.** Total biomass and ratios of multiple tissues. * indicates a *p*-value of <0.065, while not significant, is suggestive; ** indicates a significant *P*-value of <0.05. This experiment was performed with at least six plants for each genotype for each trait. For dry weight, tissue was dried for 48 hours at 60°C. To analyze the data, an ANOVA was performed using SAS9.1.3 (Cary, NC).

Table S1. Relative levels of metabolites in fruit pericarp sampled at different developmental stages in the control and L4080. Emboldened values differ (p<0.05).

Metabolite	Fold-change in L4080 vs. WT			
	14 DPA	21 DPA	42 DPA	Breaker
Alanine	14.778	0.487	1.048	5.650
Aspartate	**0.210**	0.457	0.589	2.259
Benzoate	1.855	0.535	1.006	1.250
Buturo-1,4-lactam (1TMS)	2.269	0.682	0.629	6.020
Citrate	1.112	0.604	0.883	1.107
2-Desoy-pentos-3-ylose dimethoxyamine	0.899	0.797	1.081	1.343
Erythronate	0.727	0.611	0.999	0.688
Fructofuranose 5TMS	1.118	1.865	1.360	1.574
Fructose MX1	1.050	1.121	1.405	0.794
Fructose MX2	0.515	1.000	2.382	0.110
Galactinol Put	1.078	**0.528**	1.142	1.060
Galactonate	0.629	0.712	1.032	1.136
Glucose MX1	1.223	0.905	**1.384**	1.130
Glucose MX2	1.694	0.817	2.001	0.966
Glucose-6-P MX1	1.218	0.796	0.787	3.563
Glucose-6-P MX2	1.162	0.695	0.919	0.886
Glutamine 3TMS	2.515	1.556	0.360	1.446
Glycerate	0.968	0.754	1.096	0.640
Glycerol-3-P	0.756	0.825	1.295	0.909
Glycine 3TMS	0.658	1.769	0.411	1.460
Heptadecanoate	0.813	0.600	1.614	1.013
Hexadecanoate	1.069	0.805	1.365	0.960
Inositol	0.754	0.815	1.327	1.272
Inositol-1-P	0.967	**0.402**	0.485	1.259
Isoleucine	0.359	0.618	2.678	1.006
Malate	0.659	**0.527**	0.935	0.993
Mannitol	0.048	0.843	33.337	0.012
Nonanoic acid	0.928	0.571	1.160	2.046
Norleucine	0.730	0.782	0.894	0.982
Octadecanoate	1.024	**0.769**	1.292	1.082
Oxalate	1.032	0.875	0.660	1.106
Phenylalanine	1.159	0.805	0.636	1.267
Phosphate	0.886	0.717	1.082	0.737
Pyroglutamate	0.411	**0.361**	0.421	1.814
Quinate	1.520	**0.401**	1.574	0.056

Saccharic acid 6TMS	0.559	**0.361**	0.804	1.542
Serine 3TMS	1.048	0.419	0.607	1.277
Succinate	0.628	**0.527**	**1.819**	1.541
Sucrose	0.570	**0.611**	0.586	0.760
Tetradecanoate	1.073	0.945	1.879	1.316
Tyrosine	1.825	1.024	1.188	0.311
Urea	0.898	1.169	2.157	1.192
Valine	0.379	0.639	0.666	1.215
Xylose MX1	1.136	0.885	0.807	1.438
Xylose MX2	0.807	0.789	2.730	1.852
Unknown	0.936	0.947	0.779	1.257
Unknown	1.080	0.685	0.839	2.428
Unknown	0.736	0.959	0.980	0.884
Unknown	0.766	2.929	1.648	2.444
Unknown	0.839	0.798	0.832	0.908
Unknown	0.787	**0.498**	1.027	1.765
Unknown	1.690	0.715	0.547	0.868
Unknown	0.913	0.543	**0.428**	1.153
Unknown	1.007	0.735	1.795	0.216

Table S2. **Changes in gene expression using the GeneChip® Tomato Genome Array.** This analysis was performed on leaves harvested at 12 h light (noon) in 6-week-old plants. Affymetrix ID, corresponding Genbank and Tomato Accession ID from SolGenomics network, function and Gene Ontology Annotation are given. cDNAs were listed if they varied at least 1.5-fold from the control at *p*<0.01.

Affymetrix ID	*p*-Value	Fold Change	Genbank ID	Tomato Accession (ITAG2.4)	Gene description based on ITAG2.4 ID	Function	Gene Ontology
Les.5918.1.S1_at	0	12.51	AY224079.1	Solyc01g094910.2	Ferric-chelate reductase *fro1*	Catalytic	GO:0003824
Les.1431.1.A1_at	0.001	6.75	BG631562	Solyc03g119970.2	ADP ATP carrier protein	Protein binding	GO: 0005515
LesAffx.14736.1.S1_at	0	-4.38	AW622136	Solyc11g066150.1	Bifunctional polymyxin resistance protein	Metabolic	GO:0008152
LesAffx.24134.1.S1_at	0.01	3.89	CK720539	Solyc06g082030.2	Gibberellin 2-beta-dioxygenase 7	Catalytic	GO:0003824
Les.4902.1.S1_at	0.007	3.70	BT012867.1	Solyc01g108780.2	Alpha-hydroxynitrile lyase	Catalytic	GO:0003824
Les.4488.1.S1_at	0.01	3.43	M61914.1	Solyc09g008670.2	Threonine deaminase td	Catalytic	GO:0003824
LesAffx.68994.1.S1_at	0.008	2.83	BM410725	Solyc04g014730.2	Fumarylacetoacetase	Catalytic	GO:0003824
Les.5096.1.S1_at	0.001	2.68	BT013271.1	Solyc06g073080.2	Flavonol synthase	Catalytic	GO:0003824
Les.2591.1.S1_at*	0.008	-2.25	M80608.1	Solyc01g060020.2	Beta-1,3-glucanase mRNA, complete cds	Metabolic	GO:0008152
				Solyc01g059990.2	Serine/threonine-protein phosphatase 7	None	None
LesAffx.31406.1.S1_at	0.005	-2.24	BM410312	Solyc05g005180	Similar to naphthoate synthase	Metabolic	GO:0008152
Les.5233.1.S1_at	0.001	-2.14	CN384566*	Solyc03G123620	Pectinesterase	Catalytic	GO:0003824
Les.3299.1.S1_at	0.009	2.13	BI933484	Solyc01g091170.2	ARGINASE 2 ARG2	Catalytic	GO:0003824
Les.2572.2.S1_a_at	0.004	2.02	CN385250	Solyc07g042170.2.1	Jasmonate ZIM-domain protein 3	Protein binding	GO:0005515
LesAffx.52572.1.S1_at	0.009	-1.91	BI923284	Solyc02g089540.2	CONSTANS 1 CO1 Length = 1611	Transcription Factor	GO:0003700
Les.2100.1.A1_at	0.008	-1.83	NO HITS	NO HITS	Unknown protein	None	None
AFFX-Le_GlutTrans_3_at	0.009	1.83	AAF22647	Solyc07g056480.2	Glutathione S-transferase/peroxidase	Catalytic	GO:0003824
Les.5559.1.S1_at	0.006	1.82	XP_002516460	Solyc08g065430.2.1	N-myc downstream regulated protein 3	Protein binding	GO:0005515
LesAffx.64675.1.S1_a_at	0.008	1.78	NP_194722	Solyc07g018010.2.1	(bHLH) family protein	Transcription Factor	GO:0003700
LesAffx.70226.1.S1_at	0.006	1.78	P52578	Solyc10g052500.1	Phenylcoumaran benzylic ether reductase 3	Catalytic	GO:0003824

Les.178.1.S1_at	0.004	1.75	AAA53547	Solyc07g065090.1	Polygalacturonase inhibitor protein	Signaling receptor	GO:0038023
Les.4045.1.S1_at	0.007	-1.74	N/A	N/A	N/A	None	None
Les.4044.1.S1_at	0.003	-1.73	N/A	N/A	N/A	None	None
Les.3964.1.S1_at	0.005	1.69	XP_002262775	Solyc08g006320.2	WRKY transcription factor 11	Calmodulin-binding	GO:0005516 '
LesAffx.33449.1.A1_at	0.001	-1.67	NP_181797	Solyc12g010910.1	Lipase (Fragment)	Metabolic	GO:0008152
Les.5072.1.S1_at	0.008	1.65	XP_002528534	Solyc02g068270.2	Unknown Protein (AHRD V1)	None	None
LesAffx.61012.2.S1_at	0.008	-1.63	XP_002530612	Solyc02g092040.1	LRR receptor-like serine/threonine-protein kinase, RLP	Molecular transducer activity	GO:0060089
Les.4041.1.S1_at	0.004	-1.62	N/A	N/A	N/A	None	None
Les.1287.2.S1_at	0.004	1.62	N/A	N/A	N/A	None	None
Les.2189.1.S1_at	0.003	1.62	CAB08077	Solyc04g082140.2	Pectinesterase Z94058	Metabolic	GO:0008152
LesAffx.30842.1.S1_at	0	1.61	NP_180299	Solyc10g083230.1	F-box family protein	None	None
Les.4011.3.S1_at	0.001	-1.55	N/A	N/A	N/A	None	None
Les.4418.1.A1_s_at	0	-1.55	Q08655	Solyc04g071610.2	Abscisic acid stress ripening 1 Asr1	Response to stress	GO:0006950
LesAffx.53035.1.S1_at	0.001	-1.54	NP_565781	Solyc06g068050.2	Nucleic acid binding protein Alba-related	Nucleic acid binding	GO:0003676
LesAffx.39917.1.S1_at	0.005	1.53	NP_189391	Solyc02g093430.2	Glycosyl transferase family 17 protein	Membrane	GO:0016020
Les.3575.1.S1_at	0	1.52	O04681	Solyc02g077370.1	Ethylene-responsive transcription factor 2	Regulation DNA dependent	GO:0006355
Les.4895.1.S1_at	0.005	1.52	CAB65169	Solyc05g055240.2	I-box binding factor mybi	sequence-specific DNA binding transcription factor activity	GO:0003700
Les.5748.1.S1_at	0.01	-1.51	CAO45289	Solyc05g053880.2	cDNA FLJ42396 fis clone ASTRO2001107	None	None
LesAffx.64585.1.S1_at	0.002	1.51	NP_001031122	Solyc03g006250.2	GDSL esterase/lipase	Catalytic	GO:0003824
LesAffx.71442.2.S1_at	0.01	-1.50	XP_002509949	Solyc08g082170.2	Glycoside hydrolase family 28 protein	Metabolic	GO:0008152

Table S3. Identification of the predicted *cis*-elements motifs in promoters of Dof-regulated genes. [1]*cis*-elements described by O'Mailley et al (2016), Weirauch et al. (2014) and Franco-Zorrilla et al. (2013), using the Find Individual Motif Occurrence (FIMO) software in Motif-based sequence analysis tools (MEME suite) (P < 0.05).

Predicted Regulatory *cis-Elements*[1]	Number of motifs	Number of genes	Genes
DOF	64	20	Solyc01g094910.2; Solyc11g066150.1; Solyc01g108780.2; Solyc09g008670.2; Solyc06g073080.2; Solyc03G123620; Solyc01g091170.2; Solyc07g042170.2; Solyc02g089540.2; Solyc07g056480.2; Solyc07g065090.1; Solyc08g006320.2; Solyc12g010910.1; Solyc02g092040.1; Solyc10g083230.1; Solyc04g071610.2; Solyc02g093430.2; Solyc02g077370.1; Solyc05g055240.2; Solyc05g053880.2
bHLH	38	12	Solyc01g094910.2; Solyc03g119970.2; Solyc06g082030.2; Solyc01g108780.2; Solyc09g008670.2; Solyc06g073080.2 Solyc07g042170.2; Solyc07g056480.2; Solyc07g018010.2; Solyc08g006320.2; Solyc04g082140.2; Solyc02g077370.1
MYB	33	12	Solyc01g094910.2; Solyc01g108780.2; Solyc09g008670.2; Solyc01g059990.2; Solyc03g123620.2; Solyc01g091170.2; Solyc08g006320.2; Solyc02g068270.2; Solyc02g092040.1; Solyc10g083230.1; Solyc05g055240.2; Solyc03g006250.2
NAC	21	12	Solyc09g008670.2; Solyc04g014730.2; Solyc06g073080.2; Solyc01g091170.2; Solyc07g042170.2; Solyc07g018010.2; Solyc07g065090.1; Solyc10g083230.1; Solyc04g071610.2; Solyc02g077370.1; Solyc05g055240.2
MADs	36	11	Solyc01g094910.2; Solyc01g108780.2; Solyc09g008670.2; Solyc01g059990.2; Solyc07g065090.1; Solyc08g006320.2; Solyc02g068270.2; Solyc02g092040.1; Solyc10g083230.1; Solyc04g071610.2; Solyc05g055240.2
C2H2	27	11	Solyc01g094910.2; Solyc03g119970.2; Solyc06g082030.2; Solyc04g014730.2; Solyc05g005180.2; Solyc01g091170.2; Solyc02g089540.2; Solyc08g006320.2; Solyc02g068270.2; Solyc05g055240.2; Solyc08g082170.2
G2-like	12	9	Solyc09g008670.2; Solyc06g073080.2; Solyc03g123620.2; Solyc07g065090.1; Solyc04g071610.2; Solyc02g093430.2; Solyc02g077370.1; Solyc05g053880.2; Solyc03g006250.2
HOMEOBOX	16	8	Solyc06g082030.2; Solyc09g008670.2; Solyc01g060020.2; Solyc03g123620.2; Solyc07g018010.2; Solyc04g082140.2; Solyc06g068050.2; Solyc05g053880.2
ORPHANS	8	8	Solyc01g094910.2; Solyc09g008670.2; Solyc06g073080.2; Solyc02g089540.2; Solyc07g056480.2; Solyc12g010910.1; Solyc02g092040.1; Solyc05g053880.2
AP2-EREBP	54	6	Solyc04g014730.2; Solyc01g060020.2; Solyc01g091170.2; Solyc08g006320.2; Solyc12g010910.1; Solyc04g082140.2

bZIP	24	6	Solyc01g094910.2; Solyc06g082030.2; Solyc06g073080.2; Solyc01g091170.2; Solyc07g065090.1; Solyc03g006250.2
GATA	13	5	Solyc01g060020.2; Solyc08g065430.2; Solyc07g065090.1; Solyc08g006320.2; Solyc12g010910.1
TRIHELIX	13	5	Solyc01g108780.2; Solyc04g014730.2; Solyc08g065430.2; Solyc02g092040.1; Solyc04g071610.2
WRK	9	5	Solyc11g066150.1; Solyc07g018010.2; Solyc07g065090.1; Solyc08g006320.2; Solyc10g083230.1
ERF	6	4	Solyc01g060020.2; Solyc01g059990.2; Solyc01g091170.2; Solyc08g006320.2

Table S4. Members of the *zDof* gene family with proven roles in altering metabolism and storage product accumulation. The sequence of each gene was identified, analyzed by BLAST to find the closest Arabidopsis homologue and the subclass determined.

Species	Gene	Gene role in storage product accumulation	Arabidopsis homologues	Dof Sub Group	Ref*
Soybean	*GmDof4*	Increase fatty acids and lipids content when overexpressed in soybean.	AT5G60850	A	[1]
Soybean	*GmDof4*	Enhance lipid content when expressed in *Chlorella ellipsoidea.*	AT5G60850	A	[2]
Soybean	*GmDof11*	Increase fatty acids and lipids content when overexpressed in soybean.	AT2G46590	C2.1	[1]
Maize	*ZmDof1*	Increase C and N assimilation under low N when expressed in rice.	AT1G07640	B1	[3]
Maize	*ZmDof36*	Increase in starch accumulation	AT3G45610	C2.1	[4]
Wheat	*TaDof1*	Increase expression level under low N in wheat.	AT2G37590	B1	[5]
Rice	*OsDof25*	Increase ammonium transporters level and affect N metabolism when overexpressed in Arabidopsis.	AT2G37590	B1	[[6]
Arabidopsis	*Dof1*	Increase N assimilation under low N when overexpressed in tobacco.	AT1G51700	A	[7]
Sweet	*SRF1*	Higher starch, less sugar when overexpressed in sweet potato.	AT5G39660	D1	[8]
Canola	*BnDof5.6*	Decrease fatty acid content and alter seed fatty acids composition in a *BnDod5.6* down-regulated canola line.	AT5G62940	C1	[9]
Kiwifruit	*AdDof3*	Starch degradation during fruit ripening.	AT2G37590	B1	[10]
Tomato	*AtCDF3 /AtDof3.3*	Ectopic overexpression in tomato increased fruit and vegetative biomass and improved plant response under limited and sufficient N	AT3G47500	D1	[11]
Tomato	*SlCDF4*	Overexpression in tomato increased fruit yield and vegetative biomass and improved plant response under limited and sufficient N	Not reported	D	[12]

1. Wang, H.W.; Zhang, B.; Hao, Y.J.; Huang, J.; Tian, A.G.; Liao, Y.; Zhang, J.S.; Chen, S.Y. The soybean Dof-type transcription factor genes, GmDof4 and GmDof11, enhance lipid content in the seeds of transgenic Arabidopsis plants. *The Plant journal : for cell and molecular biology* **2007**, *52*, 716-729, doi:10.1111/j.1365-313X.2007.03268.x.
2. Zhang, J.; Hao, Q.; Bai, L.; Xu, J.; Yin, W.; Song, L.; Xu, L.; Guo, X.; Fan, C.; Chen, Y. Overexpression of the soybean transcription factor GmDof4 significantly enhances the lipid content of Chlorella ellipsoidea. *Biotechnology for biofuels* **2014**, *7*, 128.
3. Kurai, T.; Wakayama, M.; Abiko, T.; Yanagisawa, S.; Aoki, N.; Ohsugi, R. Introduction of the ZmDof1 gene into rice enhances carbon and nitrogen assimilation under low-nitrogen conditions. *Plant Biotechnology Journal* **2011**, *9*, 826-837.
4. Wu, J.; Chen, L.; Chen, M.; Zhou, W.; Dong, Q.; Jiang, H.; Cheng, B. The DOF-Domain Transcription Factor ZmDOF36 Positively Regulates Starch Synthesis in Transgenic Maize. *Frontiers in Plant Science* **2019**, *10*, doi:10.3389/fpls.2019.00465.

5. Kumar, R.; Taware, R.; Gaur, V.S.; Guru, S.K.; Kumar, A. Influence of nitrogen on the expression of TaDof1 transcription factor in wheat and its relationship with photo synthetic and ammonium assimilating efficiency. *Molecular biology reports* **2009**, *36*, 2209-2220, doi:10.1007/s11033-008-9436-8.
6. Santos, L.A.; de Souza, S.R.; Fernandes, M.S. OsDof25 expression alters carbon and nitrogen metabolism in Arabidopsis under high N-supply. *Plant biotechnology reports* **2012**, *6*, 327-337.
7. Wang, Y.; Fu, B.; Pan, L.; Chen, L.; Fu, X.; Li, K. Overexpression of Arabidopsis Dof1, GS1 and GS2 enhanced nitrogen assimilation in transgenic tobacco grown under low-nitrogen conditions. *Plant Molecular Biology Reporter* **2013**, *31*, 886-900.
8. Tanaka, M.; Takahata, Y.; Nakayama, H.; Nakatani, M.; Tahara, M. Altered carbohydrate metabolism in the storage roots of sweetpotato plants overexpressing the SRF1 gene, which encodes a Dof zinc finger transcription factor. *Planta* **2009**, *230*, 737-746.
9. Deng, W.; Yan, F.; Zhang, X.; Tang, Y.; Yuan, Y. Transcriptional profiling of canola developing embryo and identification of the important roles of BnDof5.6 in embryo development and fatty acids synthesis. *Plant & cell physiology* **2015**, *56*, 1624-1640, doi:10.1093/pcp/pcv074.
10. Zhang, A.D.; Wang, W.Q.; Tong, Y.; Li, M.J.; Grierson, D.; Ferguson, I.; Chen, K.S.; Yin, X.R. Transcriptome Analysis Identifies a Zinc Finger Protein Regulating Starch Degradation in Kiwifruit. *Plant Physiol* **2018**, *178*, 850-863.
11. Domínguez-Figueroa, J.; Carrillo, L.; Renau-Morata, B.; Yang, L.; Molina, R.V.; Marino, D.; Canales, J.; Weih, M.; Vicente-Carbajosa, J.; Nebauer, S.G.; et al. The Arabidopsis Transcription Factor CDF3 Is Involved in Nitrogen Responses and Improves Nitrogen Use Efficiency in Tomato. *Front Plant Sci* **2020**, *11*, 601558, doi:10.3389/fpls.2020.601558.
12. Renau-Morata, B.; Carrillo, L.; Cebolla-Cornejo, J.; Molina, R.V.; Martí, R.; Domínguez-Figueroa, J.; Vicente-Carbajosa, J.; Medina, J.; Nebauer, S.G. The targeted overexpression of SlCDF4 in the fruit enhances tomato size and yield involving gibberellin signalling. *Sci Rep* **2020**, *10*, 10645, doi:10.1038/s41598-020-67537-x.
